# AI based optimization of injection pressure for hydrogen and spirogyra biodiesel dual fuel engine to enhance combustion performance and emission characteristics

**DOI:** 10.1038/s41598-025-34179-w

**Published:** 2026-02-10

**Authors:** S. Aravind, Debabrata Barik, Prabhu Paramasivam, Dhinesh Balasubramanian, Utku Kale, Artūras Kilikevičius

**Affiliations:** 1 Department of Mechanical Engineering, Karpagam Academy of Higher Education, Coimbatore, 641021 India; 2 Centre for Energy and Environment, Karpagam Academy of Higher Education, Coimbatore, 641021 India; 3 Centre of Research Impact and Outcome, Chitkara University, Rajpura, Punjab 140401 India; 4 Department of Port Engineering, Lithuanian Maritime Academy (LMA), Vilnius Gediminas Technical University, Klaipėda, Lithuania; 5 Department of Aeronautics and Naval Architecture, Faculty of Transportation Engineering and Vehicle Engineering, Budapest University of Technology and Economics, Műegyetem rkp. 3., Budapest, H-1111 Hungary; 6 Mechanical Science Institute, Vilnius Gediminas Technical University, Plytinės g. 25, Vilnius, 10105 Lithuania

**Keywords:** Sustainable biofuels, Hydrogen energy, AI-Driven calibration, Renewable energy, Machine learning for biofuels, Eco-Friendly fuel optimization, Energy science and technology, Engineering

## Abstract

The principal objective of this research is to employ modern machine learning techniques to optimize high-pressure biofuel injection strategies for sustainable energy applications. An engine powered with biofuel and hydrogen (H₂) under dual-fuel (DF) mode was tested under a varied fuel injection pressure range from 180 to 240 bar for optimization and modeling. The results demonstrate that an injection pressure of 220 bar produces enhanced engine performance. At this pressure, enhancements were noted in combustion characteristics, efficiency, and emission levels. The ignition delay (ID) at 220 bar injection pressure was 9.4% longer than at 240 bar injection pressure. The 220 bar IP mix demonstrated reduced peak cylinder pressure (PCP) and heat release rate (HRR) compared to the 240 bar. A 12.4% rise in brake-specific fuel consumption (BSFC) was observed at 220 bar inlet pressure. Nevertheless, although brake thermal efficiency (BTE) increased with increasing injection pressure (IP), the increase at 220 bar was somewhat less than that at 240 bar. Despite elevated nitrogen oxide (NOx) emissions with the 220 bars compared to pure diesel, carbon monoxide (CO) and hydrocarbon (HC) emissions were markedly decreased. Smoke emissions were reduced with the 220 bars in comparison to diesel and other fuel combinations. Three machine learning models were employed to establish a predictive control framework. The decision tree (DT) model had the greatest accuracy, with R² values of 0.9792 for PCP and 0.9710 for HC, alongside near-zero MAPE for BTE and HC This study underscores the potential of AI-driven biofuel optimization for fostering sustainable transportation and renewable fuel strategies, paving the way for large-scale adoption of low-carbon, high-efficiency energy solutions.

## Introduction

In order to provide sustainable and eco friendly energy system, many researchers and engineers investigated on alternative fuels and advanced engine technology^1,2^. In a result an innovative idea which use the combination of algae biodiesel in a dual fuel (DF) mode on diesel engines^3^. This method alleviates the boundaries of traditional diesel combustion while using the advantages of the algae-based biodiesel as a renewable and huge availability of the resources^4^. To increase the efficiency of the DF engines, optimization of the injection pressure (IP) is very important as its variation significantly affects combustion efficiency, emissions, and overall performance rating of the engine^5^. To find the optimal IP values will ensure the condition for proper ignition and combustion of algal biodiesel mixture by methodical testing and analysis^6^. Studying DF diesel engines with biodiesel provides solution for improving the energy efficiency and usage of sustainable energy^7^. This alternative method reduce the effects on environmental pollution and provides the way for usage of non-renewable energy sources with alternative fuels^8,9^.

Singh et al.^10^ investigated the feasibility of using chlorella algae biodiesel in direct-injection diesel engines. They performed experiments using B20 chlorella algae methyl ester at varying injection pressures to assess combustion efficiency and emission properties. Their research emphasized the need to attain suitable IP for improved mechanical and combustion performance. Rajak et al.^11^ inspected the effectiveness of microalgae spirulina biodiesel in diesel engines, assessing B0, B10, B20, and B50 blends at IP ranging from 18 to 26 MPa. Their results showed that B20 at 22 MPa decreased NOx and smoke emissions while conserving engine performance, highlighting the feasibility of microalgae biodiesel as a valid alternative fuel. Tatikonda et al.^12^ examined the effect of IP on engine performance with methyl ester derived from Chlorella vulgaris microalgae. Their research outcome showed a slight effect on BSFC but an improvement in BTE at 200 bar IP, emphasizing the requirement for IP modification.

Selvaraj et al.^13^ studied the influence of fuel IP on engine performance and emissions using frying oil biodiesel. Their experimental results showed increased BTE and reduced emissions, showing the test biodiesel as an eco-friendly alternative for fossil fuels. Sharma et al.^14^ studied about the combustion properties of biodiesel with polenta oil mixtures. The B20 blend showed an improvement in performance when injected at 31° bTDC and IP of 240 bar, resulting in improved efficiency and reduced emissions. Mulayalu et al.^15^ measured the performance of a DI CI engine using shea olein methyl ester-diesel blends, indicating that an IP of 210 bar enhanced engine performance and reduced emission level.

Sarıdemir et al.^16^ explored the influence of maize oil methyl ester blends on combustion, performance, and emissions characteristics in diesel engines. They observed that raised IP improved combustion efficiency and reduced CO and HC emissions, although it showed an increment in the NOx concentrations. Kumar et al.^17^ studied the influence of cerium oxide nanoparticles on biodiesel obtained from waste cooking oil, showing increase in IP (180, 210, and 240 bar) improved combustion efficiency and decrement on the emission level. Shrivastava et al.^18^ in their experimental work carried out using Roselle biodiesel blends at varying iP, indicating that an IP of 220 bar with RB20 showed best performance and reduced CO_2_ emissions. Kim et al.^19^ examined the effect of IP on diesel engine combustion and emissions with palm oil biodiesel, showing an raised IP at low speeds reduction in ignition delay, enhanced combustion efficiency, and reduced soot emission. However, found an increased NOx emission, although decrease in CO and HC were insignificant. The effects were upturned using 100% palm biodiesel.

Karikalan et al.^20^ investigated performance and emissions of diesel engine with different biodiesel IP, demonstrating the feasibility of biodiesel use in CI engines without considerable alterations. Yesilyurt et al.^21^ inspected the influence of fuel IP on diesel engines using waste cooking oil biodiesel blends, identifying that 210 bar is perfect for better torque, BP, BTE, and reduced HC and smoke emissions. However, increase in the emissions of NOx and CO_2_ were noted. Arunprasad et al.^22^ studied the effects of IP and time on the effectiveness and emissions of CI engines using biodiesel blends, their experimental findings showed increase in IPs improved BTE and decreased emissions, while increased NOx and CO_2_ emission levels.

Yoon et al.^23^ evaluated the combustion and emission characteristics of a CRDi diesel engine using diesel-palm biodiesel mixes. Their results showed that increase in IP improves combustion, HRR, and IMEP, while BSFC, HC, and particulate matter showing a declining trend. However, emissions of CO and NOx increased. Rajak et al.^24^ investigated the effect of IP on CI engines using microalgae biodiesel blends, indicating that 22 MPa IP with B20 decreased EGT and particulate matter emissions by 22.9%, along with minor rise in specific fuel consumption. Saravanan et al.^25^ inspected diesel engines operating with pine methyl ester, identifying that rise in IP improved BTE and combustion while reducing CO, HC, and smoke emissions, anyhow found an increased Nox emission level. Anas et al.^26^ performed experiment to study the impact of IP on ID and emissions for several biodiesel types, indicating that raised IPs declined ID while increasing CO_2_ emissions due to improved atomization and combustion efficiency.

Jagadish et al.^27^ studied CI engines operating on biodiesel blends, showing that decreased IP improved the BSFC and BTE, while increased IP increased NOx and HC emissions. Vasavi A^28^ emphasized the need for alternate fuels and examined algal biodiesel in CI engines at different IP level. Their results showed that an inlet pressure of 220 bar resulted in improved performance characteristics, showing the effectiveness of mid-level inlet pressure.

Hydrogen has showed the capability of improving the combustion efficiency and reduced emission level of CI engines. Enhancing combustion efficiency along with reduction in emission level imposes understanding of the intricate liaisons amid engine settings, fuel characteristics, and environmental factors^29,30^. Conventional experimental models often neglect to account for complicated associations amongst these factors. Machine learning (ML) techniques deliver a feasible substitute by detecting nonlinear relationships and veiled patterns that conservative methods fail to diagnose^31,32^. Through the analysis of wide-range of datasets including engine parameters, fuel characteristics, and emissions data, ML techniques permit the formation of exact forecasting models intended at improvement in engine performance and minimalizing the environmental consequences^33,34^.

Furthermore, ML models are adaptable and change over time by integrating new datasets, hence enhancing prediction accuracy^35^. Subsequently, the application of ML in engine performance and emissions modeling has substantial potential for progressing environmentally sustainable conveyance technologies and justifying the ecological problems related to IC engines^36,37^. The current research work studies the influence of IP on emissions and combustion in CI engines using biodiesel blends, highlighting the implication of IP optimization in improvising the performance characteristics. Also, it examines approaches to advance combustion efficiency in CI engines, along with nano-additives, and cetane boosters. Advanced ML methods, like decision trees and random forests, will be used to make predictive models. A baseline linear regression machine learning technique will be used for model valuation and contrast.

Recent investigations has shown that coupling artificial intelligence with combustion modeling improvise the predictive precision and optimization possibilities in engine studies. For example, Taghavifar et al.^38–40^ efficiently combined the computational fluid dynamics (CFD) with artificial neural networks (ANNs) to forecast emission characteristics, combustion characteristics, and wall heat flux in diesel engines with outstanding precision (R² ≈ 0.97–0.99). These research works found the efficiency of AI-assisted backgrounds in apprehending nonlinear combustion behaviour. Also, other research work done by Ramalingam et al.^41,42^ revealed that ammonia–biofuel and hydrogen-enriched fifth-generation biofuel blends can effectively improve the BTE, and decrement in the emissions of NOₓ and CO₂. The outcome of these studies shows a hopeful possibility of linking alternative fuel technologies with AI-based optimization to attain cleaner, highly efficient, and ecologically sustainable engine operations. Based on this groundwork, the current research work covers AI-driven modelling to find optimal IP in a hydrogen–Spirogyra biodiesel dual-fuel engine, moving forward for a sustainable energy usage and effective biofuel consumption.

## Materials and methodology

### Fuel preparation

Table 1 shows the fuel properties of diesel fuel and other test fuels as per the a contrast ASTM standards. Altering the proportions of Di-tert-butyl Peroxide (DTBP), Algae residual carbon nanoparticle (ARCNP), and Hydrogen in the biodiesel blend-1 (Spirogyra Biodiesel 30% blend with 70% diesel by volume) (SBD30), resulting in the variations of its properties. Increasing the H_2_ level improves the CV and cetane number, enhancing the ignition characteristics^43–45^. The objective of this work is to evaluate the influence of the fuel blend on combustion efficiency, engine behavior, and emissions to produce perceptions for optimizing engine performance and reducing environmental effects. For an additional inclusive knowledge on the creation, preparation process, and preceding authentication of this specific fuel mixture SBD30 + 2%ARCNP + 1.5%DTBP + 15H_2_, readers are suggested to refer to the cited reference^46^, which covers the detailed experimental procedures and characterization data supporting the present blend composition.

**Table 1:** Fuel properties.

Properties	ASTM	Diesel only	SBD30 (blend 1)	SBD30 + 2%ARCNP + 1.5%DTBP + 15H_2_ (Blend 1)
Cetane number	D 613	48.9	50.56	60.31
Density (kg/m^3^) @ 15 °C	D 4052	823.78	843.99	838.89
Flash point (°C)	D 93	51.9	64.89	64.1
CV (MJ/kg)	D 4809	45.64	44.49	65.89
Kinetic viscosity @ 40 °C, (mm^2^/s)	–	3.99	4.215	3.81

### Test setup

The system comprises of a cylinder filled with hydrogen, measuring device for hydrogen, and an accurate injection device employing a 5-hole solenoid-controlled fuel injector. The hydrogen cylinder is incorporated with flame trappers and arresters to ensure the safety of the system.

**Fig. 1:**
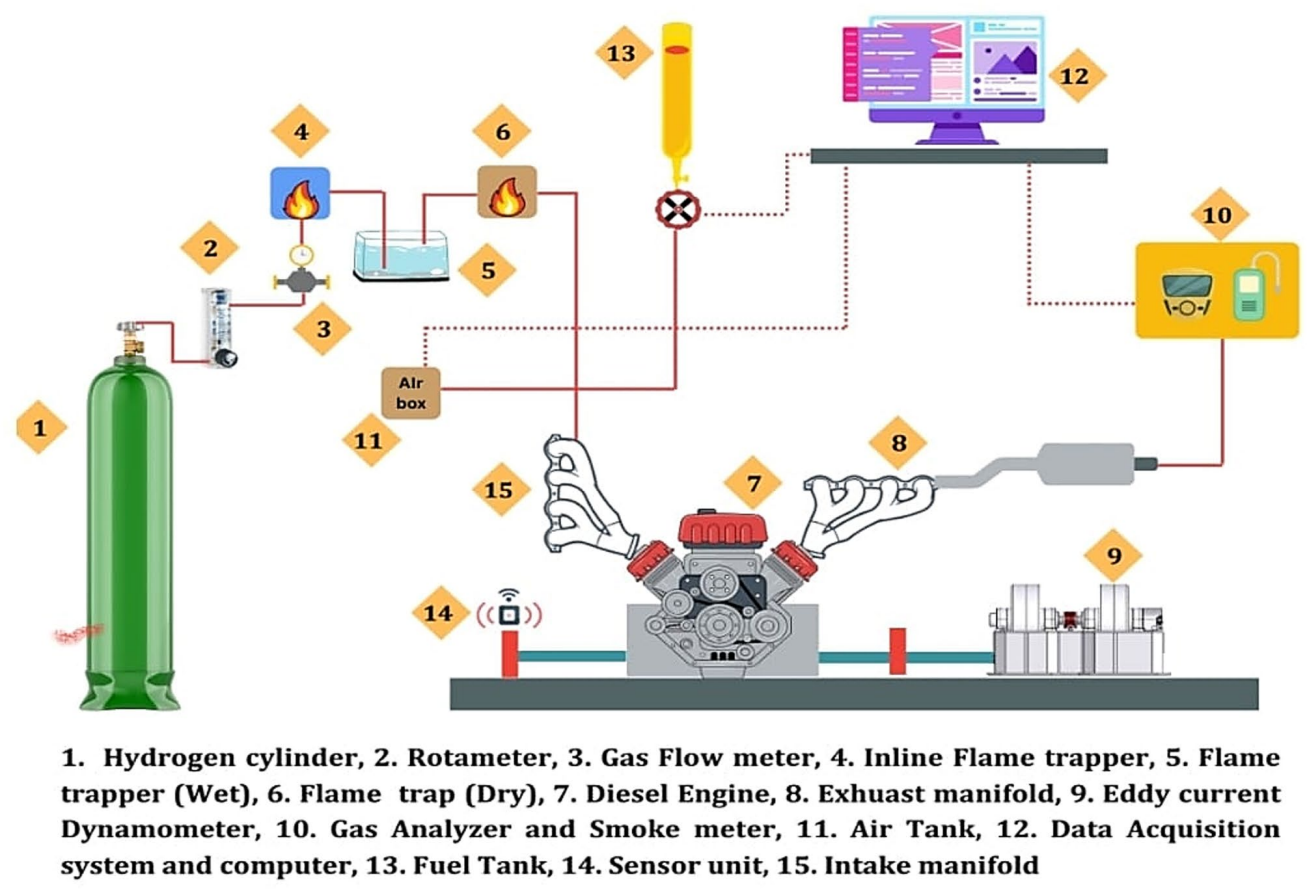
Schematic layout of hydrogen injection setup.

**Fig. 2:**
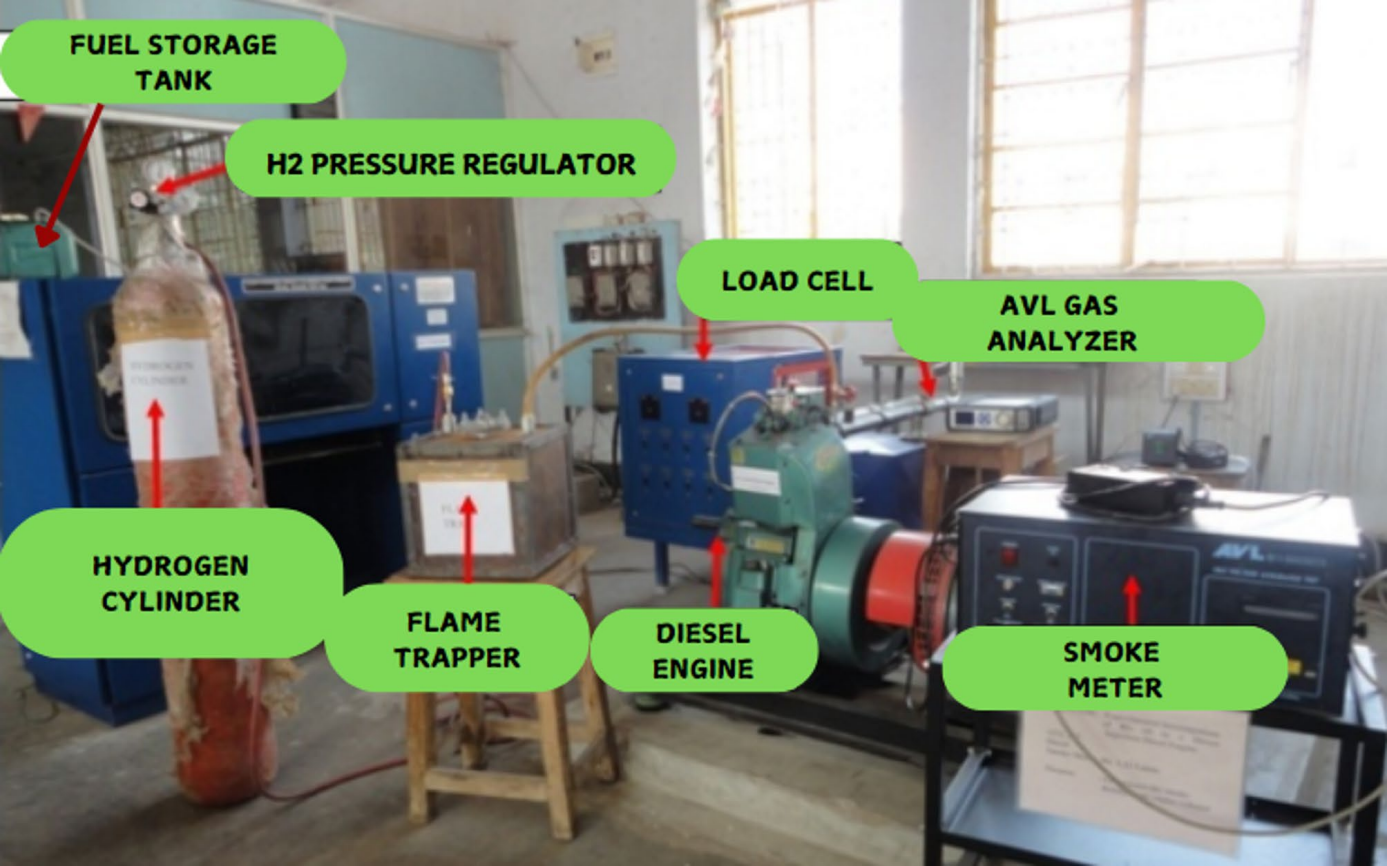
Actual test setup.

A Flame trap was utilized to prevent flames from returning back from the supply system^47^. These safety procedures are vital when working with such highly flammable H_2_ gas as shown in Fig. 1. The picture of the test setup is showcased in Fig. 2. Injection emerges next to the intake valve through an electronically controlled injector furnished with a solenoid mechanism and five separate orifices, each owning a nozzle (dia. = 0.126 mm). An Atmega-328 microprocessor administers the injection process by regulating the hydrogen (H_2_) supply with the help of PWM to control the fuel injection volume according to recognized criteria. The L293D adapter gives the essential power supply to the injector, pump, and microcontroller, giving a precise power supply and synchronization for all the system components.

This engine particularly has a constantly variable compression ratio using cautiously engineered portable chamber blocks, conserving the controls of the ignition chamber. Critical device measures the cylinder pressure and crank angles, imparting the information to the PC for the construction of Pθ − PV charts. Measurement devices for airflow, fuel flow, temperature, and load comprises of manometers and transmitters. A PLC-controlled arrangement controls the flow of hydrogen and cetane boosters. The flow rates of cooling water, along with calorimeter water, are measured using two rotameters. The configuration is connected with LabVIEW-based “ICEnginesoft,” for real-time controlling and assessment. Table 2 explains the complete specs of the test setup.

**Table 2:** Engine specification.

Model of engine	Kirloskar TV1
Manufacturer	Kirloskar India
Cooling	Water
Type	4-Stroke, single cylinder, diesel
Bore and stroke	0.0875 m × 0.11 m
Cubic capacity	0.661 l
Compression ratio	17.5
Operating speed range	750 RPM to 2000 RPM
Minimum operating speed	1200 RPM
Fuel injection timing	23° bTDC
Inlet valve clearance	0.018 m
Exhaust valve clearance	0.02 m
Lubrication system	Forced feed system

#### Uncertainty analysis

All the measuring devices in engine test setup are subject to intrinsic mistakes, making fault analysis vital for data consistency. For example, digital gas analysers may show erroneous readings due to sensor deprivation, while dynamometers can misrepresent torque or speed, affecting brake power approximation. Similarly, crank angle encoders and speed sensors may experience signal delays, and smoke meters can produce improper opacity values due to environmental effects. Fuel burettes are delicate to vibration and physical handling, and pressure transducers may be spoiled by variation in temperature and response of the dynamic conditions. These errors may potentially affect the accuracy level of the experimental data’s if they are not properly calibrated and corrected.

The Uncertainties resulting from the major errors in the physical quantities are found using the general equation, which is given below^48^.

$$\:\frac{{E}_{y}}{y}=\:\sqrt{\left[\sum\:_{i=1}^{m}{\left(\frac{1}{y}\frac{\partial\:y}{\partial\:{z}_{i}}\right)}^{2}\right]}$$

In this above relation, each variable $\:y$ signifies a physical quantity that depends on numerous parameters $\:{z}_{i}$, whereas $\:{E}_{y}$means the overall uncertainty related to the measurement. This relationship helps to enumerate the spread of discrete measurement errors within the experimental setup. The specific measurement uncertainties of all devices utilized in this current study are briefed in Table 3.

**Table 3:** Measurement uncertainty and instrumentation analysis.

Instrument	Parameters measured	Range	Accuracy	Uncertainty (%)
Gas analyzer	NO emissions	0–5000 ppm	± 10 ppm	± 0.2
	CO emissions	0–10%	± 0.02%	± 0.15
	HC emissions	0–10,000 ppm	± 20 ppm	± 0.2
	CO2 emissions	0–20%	± 0.03%	± 0.2
Dynamometer	Brake power	0–100 Nm	± 0.1 Nm	± 0.2
Crank angle encoder	Crank position	0–720°	± 0.2 °CA	± 0.2
Speed sensor	Engine speed	0–10,000 rpm	± 10 rpm	± 0.1
Smoke meter	Smoke opacity	0–100%	± 0.1	± 1
Burette	Fuel consumption	0–100 ml	± 0.1 cc	± 1
Pressure transducer	In-cylinder pressure	0–100 bar	± 0.1 bar	± 0.15

### Injection pressure variation method

To vary the IP in a test setup of an SC diesel engine working in a DF mode, a pressure controller is used in the fuel supply line. The pressure controller is a critical component that allows precise manipulation of the IP. Operators can dynamically adjust the fuel pressure fed to the injector nozzle by manually operating the pressure controller. This approach assures the ability to alter and house various operational conditions, for optimizing combustion efficiency, emissions, and overall engine performance. The pressure controller functions as a dynamic mechanism that can be varied precisely using real-time data from the sensors, which offers a practical and efficient method to get the anticipated IP in DF mode without demanding complicated modifications to the fuel system.

### Engine performance

#### Combustion characteristics

The ID denotes the interval of time between the beginning of fuel injection and the start of combustion, showing an important role in assessing the combustion efficiency and emission characteristics. A continued ID may lead to either premature or late combustion. Attaining optimal combustion duration also confirms efficient energy release^49^. The variation in cylinder pressure with respect to the crank angle provides perceptions into pressure variations during the engine cycle, helping in evaluating combustion performance and optimizing policy for fuel injection for maximum power output^50^. The heat release rate quantifies the rate of energy release during combustion, offering valuable data on combustion dynamics, aiding in refining injection strategies, and inducing overall engine efficiency^51^.

#### Performance attributes

BSFC is considered as a gauge of an engine’s fuel economy, representing the volume of fuel consumed per unit of power output. A lower BSFC value signifies improved efficiency in fuel consumption^52^.

1 $$\:BSFC=\frac{FFR\:}{BP},\frac{g}{kWhr}$$

BTE evaluates the efficacy of an engine in translating fuel energy into useful mechanical yield. Higher BTE values show a better overall efficiency and improved energy consumption^53^.

2 $$\:BTE=\frac{BP\times\:3600}{FFR\times\:CV}\times\:100\:\%$$

EGT measures the residual thermal energy in exhaust gases post-combustion. Monitoring EGT is vital to avoid extreme temperatures and optimize air-fuel mixing.

#### Emission characteristics

Carbon monoxide emissions are due to poor combustion, which highlights inefficiencies in the combustion process. Drop in CO levels is indispensable for both environmental sustainability and public health.

3 $$\:CO\:=\frac{\left(\frac{{m}_{f}+m{}_{gf}+{m}_{a}}{29}\right)\times\:10\times\:CO\:\left(in\:\%vol\right)\times\:28}{BP}\:\:\left(g/kWh\right)$$

HC emissions start from unburned hydrocarbons or partly combusted fuel. Monitoring HC levels is significant for upholding air quality and reducing pollution.

4 $$\:HC\:=\frac{\left(\frac{{m}_{f}+m{}_{gf}+{m}_{a}}{29}\right)\times\:10\times\:HC\:\left(in\:\%vol\right)\times\:13}{BP}\:\:\left(g/kWh\right)$$

NOx emissions, mainly nitrogen oxides, are principally prejudiced by higher combustion temperatures. It is measured as:

5 $$\:NO\:=\frac{\left(\frac{{m}_{f}+m{}_{gf}+{m}_{a}}{29}\right)\times\:10\times\:NO\:\left(in\:\%vol\right)\times\:32.4}{BP}\:\:\left(g/kWh\right)$$

Smoke emissions specify presence of particulate matter, which results in inefficient combustion. Minimizing smoke generation improves the quality of air, thus conforming to environmental compliance.

## Machine learning

### Linear regression

The relationship between a dependent variable and their single or multiple variables is modeled by the basic machine learning technique known as linear regression (LR). Assuming a linear type relationship among variables, it predicts the dependent variable as a linear combination of the independent variables^54^. To minimize the total squared discrepancies between the observed and predicted values, coefficients for each independent variable must be estimated during the training process. Notable in particular is the high appreciation for the interpretability, computational efficiency, and simplicity of use of linear regression. It is therefore a great option when doing jobs like predictive modeling and determining variable correlations. It is an excellent choice for measuring and comparing the baseline performance of ML^55^.

### Decision tree

Non-parametric supervised learning models called decision tree (DT) find use in both regression as well as classification applications. Employing a decision node and leaf node structure similar to a tree, DTs partition the feature space into regions depending on the feature values. To reduce errors and maximize information gain, the model chooses the feature at each decision node that splits the data into the best homogeneous subsets. Decision trees can manage non-linear relationships and are easily interpreted^56^. But, especially in deep trees, they are inclined to overfitting, which can be barred with techniques like pruning. The efficacy and easiness of usage of decision trees have made them more and more prevalent in recent years. To address specific difficulties and boost speed, researchers have used several alterations and developments to traditional decision trees. Through the use of the variety of different trees, these ensemble techniques minimize overfitting and capture complex patterns in data^57^.

Decision trees are also a constituent of more sophisticated ML approaches, where they help as basic learners or as components of more intricate models. Excluding the demerits of the decision trees their sensitivity to noisy data and their inclination to produce biased models when specific variables predominate in the splits. Scientists are still trying to find solutions for these problems via algorithmic developments, data pretreatment techniques, and feature selection. Ultimately, decision trees continue to be a valuable instrument in the ML toolbox because they offer a good mix of interpretability and predictive power^58^.

### Random forest

Random Forest is a type of ensemble learning that, in order to improve forecast accuracy and prevent overfitting, integrates many decision trees into a single machine. It does this by independently constructing a huge number of decision trees, each of which utilizes a random selection of features and is trained on a random subset of the training data^59,60^. By averaging (in the case of regression) or voting (in the case of classification), the output of each tree is compiled during the prediction process to arrive at the ultimate forecast. When compared to individual decision trees, Random Forests are very robust, they can handle high-dimensional data effectively, and they are less likely to overfit^61^. For classification and regression problems, they are utilized extensively across a wide range of fields. Because of its capacity to deliver accurate forecasts while simultaneously minimizing the risk of overfitting, this method has gained popularity. As a result, it provides a workable answer to a wide variety of real-world issues that arise in sectors such as energy, ecology, healthcare, and finance.

## Results and discussion

The present investigation is carried out to find the best IP for the H_2_ DF operation in the existing SC DI Diesel engine. In this study, the optimal fuel blend “blend-2” (BD30 + 1.5%DTBP + 2%ARCNP + 15H_2_) is tested at varying IPs ranging from 180 bar to 240 bar in steps of 20 bar. This study analyzed the engine’s combustion, performance, and emission characteristics to identify the optimal IP.

### Combustion characteristics

#### Ignition delay

**Fig. 3:**
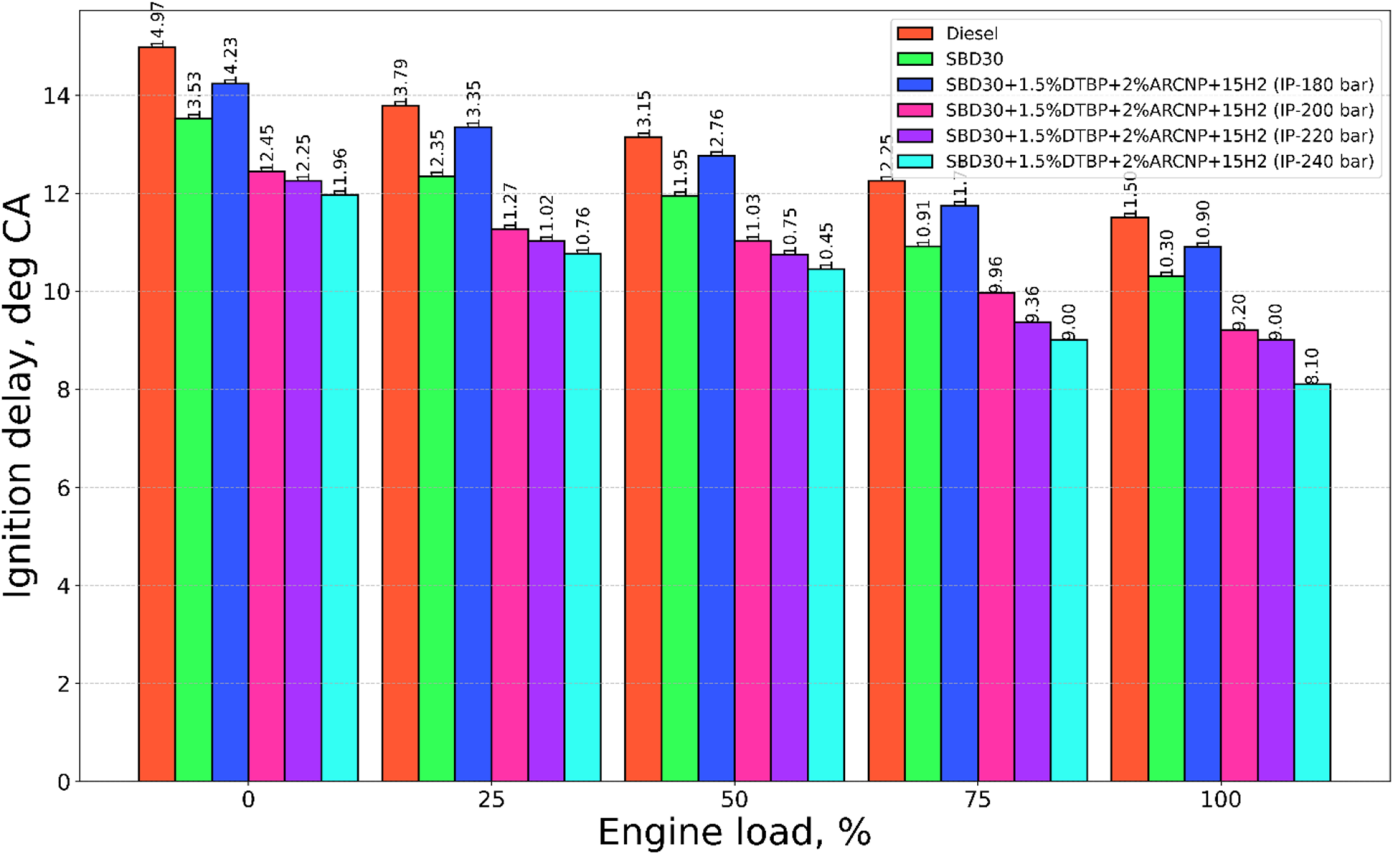
ID vs. engine load.

The ignition delay (ID) for test fuels Diesel, Blend-1, Blend-2, and optimum H_2_ flow rate of 15 LPM with varying IP of 180, 200, 220, and 240 bar is depicted in Fig. 3. The ID duration depends on various parameters when fuels like Blend-1 with DTBP and ARCNP with H_2_ are used such as volatility of the fuel, Cetane number of the fuel, IP of the fuel, and auto ignition temperature of the fuel. In the present investigation, the ID in case of all test fuels reduced at elevated engine loading. This is attributed to elevated temperature of the cylinder and the compressed air before the injection of the fuel^62,63^. This makes the fuel evaporate quickly leading to auto ignite and reducing the ID period^38^. In addition to this among all the test fuel combinations diesel gives a higher ID over the entire range of loading and it is attributed to low ignition energy. With the addition of DTBP + ARCNP + H_2,_ the fuel improves its combustion nature however it is not able to give the best output at the fixed IP of 200 bar set by the manufacturer.

With variation in IP Blend-2 (IP = 240 bar) gives the lowest ID, this is due to the superior quality of spray and the presence of high Cetane index for Blend-1 combined results for ID elevation. The ID for Blend-2 (IP = 220 bar) is 8.96°CA at 100% engine load, almost 9.4% greater to Blend-2 (IP = 240 bar).

#### Combustion duration

**Fig. 4:**
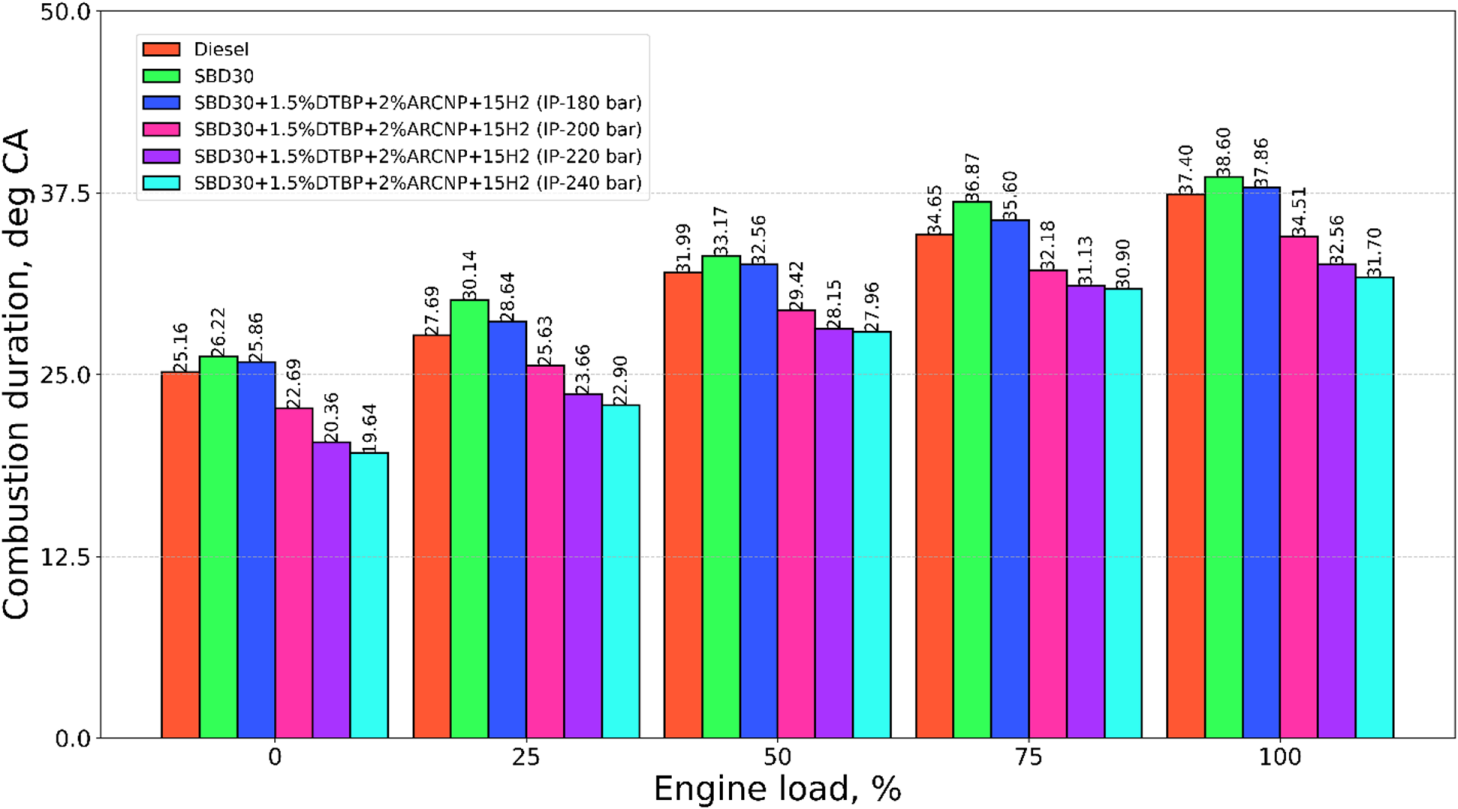
Combustion duration vs. engine load.

The combustion duration (CD) for test fuels Diesel, Blend-1, Blend-2, and optimum H_2_ flow rate of 15 LPM with varying IP of 180, 200, 220, and 240 bar is illustrated in Fig. 4. The CD for Blend-1 is higher compared to other fuels because of its low-burning nature on the other side the high-burning nature of gaseous H_2_ may lead to shorter CD^64,65^. The CD for Blend-1, Blend-2 with different IPs shows an increasing pattern of CD at higher loads, owing to higher quantity of injected fuel into the engine cylinder to adjust in accordance with speed and load^39^. However, among all these IPs, an IP of 220 bar shows a slight increase in CD in comparison to an IP of 240 bar. The CD for the IP:220 bar combination is 32.56°CA at full engine load. However, at the IP of 240 bar, the CD is 0.86°CA less compared to IP of 220 bar combination.

#### Peak cylinder pressure

**Fig. 5:**
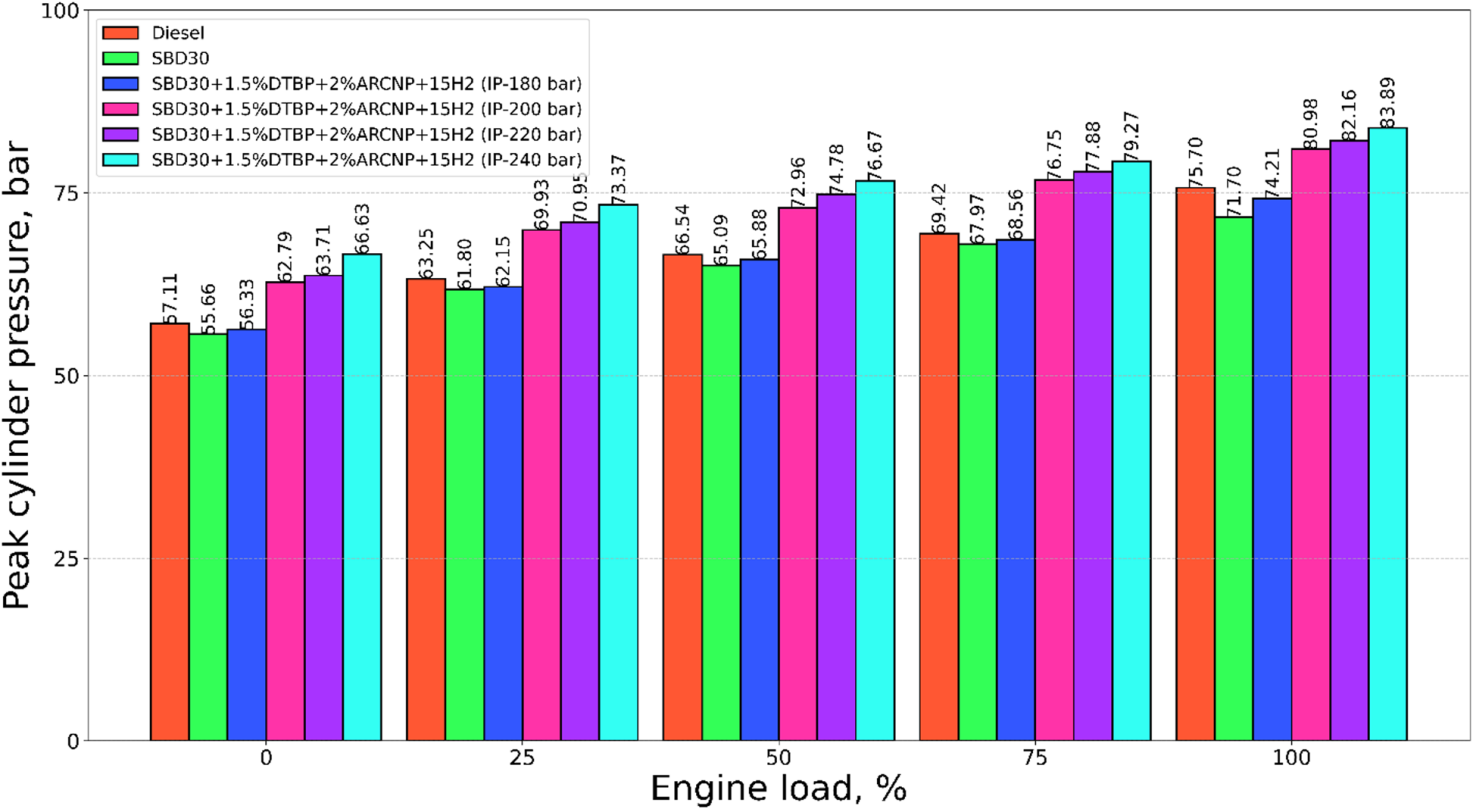
PCP vs. engine load.

Figure 5 depcits the variation of peak cylinder pressure (PCP) for all test fuels with varying IP of 180, 200, 220, and 240 bar with varying engine load. The rise in cylinder pressure is an indication of fuel volatility and ID and type of fuel used (liquid or gaseous). In the present work, PCP was observed for pilot injection of 240 bar, which is about 83.8 bar at full load. This is followed by IP = 220 bar with a value of 82.2 bar at full load. This substantial rise in PCP may be due to high-pressure injection of the fuel, which in turn converts to very fine droplets, leading to high evaporation and superior mixing with the air in the combustion chamber leading to rapid combustion, making MCP higher^65,66^. Blend-1 offers extremely lower PCP, making a smooth combustion for the test loads making almost very low vibration and deviation to the PCP data. The PCP for diesel is 75.7 bar at full load condition.

#### Cylinder pressure

**Fig. 6:**
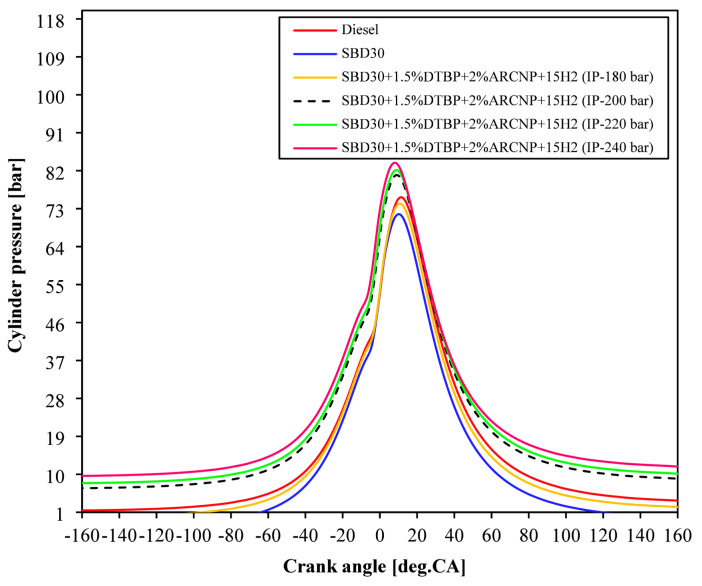
Cylinder pressure vs. °CA.

The cylinder pressure variation in the case of Diesel, Blend-1, Blend-2 with varying IP of 180, 200, 220, and 240 bar is displayed in Fig. 6. As discussed in the previous sections, the injection of H_2_ increases the combustion phenomenon in the combustion chamber. However, this is possible only when the pilot-injected fuel ignites quickly, leading to a rise in pressure. For this purpose, the IP of the engine was considered from 180 bar to 240 bar. Among all, the pea cylinder pressure was noticed for Blend-2 (IP = 220 bar) and Blend-2 (IP = 240 bar). In addition to this, due to high-pressure injection, the ID becomes shorter, making a smooth pressure reduction in the diffusion combustion stage. This leads to an increased lifespan of the engine. The peak cylinder pressure for diesel, Blend-1, Blend-2 (IP = 180 bar), Blend-2 (IP = 200 bar), Blend-2 (IP = 220 bar), and Blend-2 (IP = 240 bar) occurred at 11.5°CA, 10.3°CA, 10.9°CA, 9.2°CA, 9.0°CA, and 8.1°CA at full load respectively.

#### Heat release rate

**Fig. 7:**
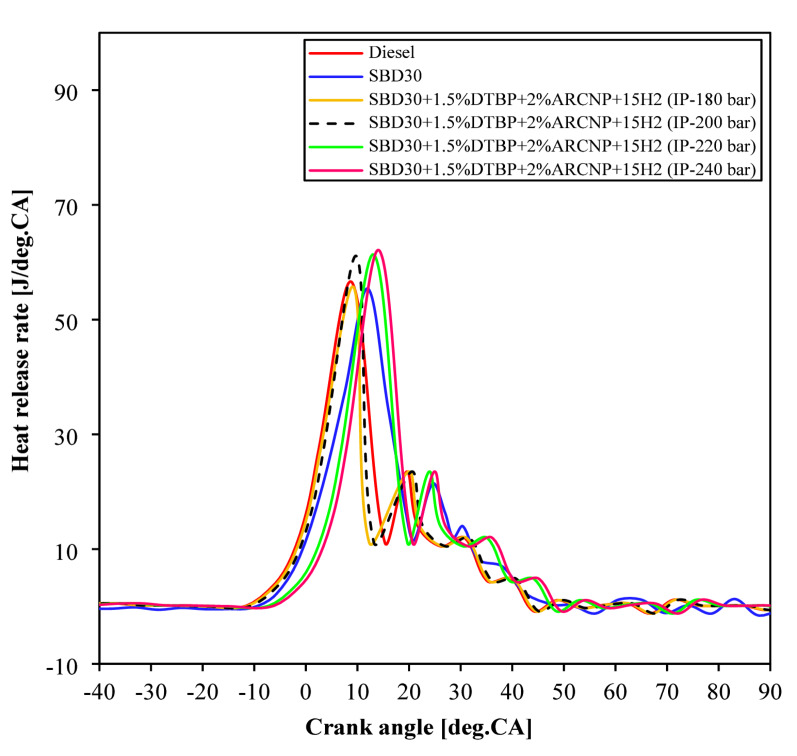
HRR vs. °CA.

Figure 7 shows the net HRR of Diesel, Blend-1, Blend-2with varying IP of 180, 200, 220, and 240 bar for varying crank angles. The HRR represents the amount of heat released in the combustion process from combustion chamber at each degree of crank angle. The maximum HRR is achieved for Blend-2 (IP = 240 bar) of 62.0 J/°CA, followed by Blend-2 (IP = 220 bar) of 61.25 J/°CA, at 9.01°CA and 8.15°CA, correspondingly. This higher HRRs for the Blend-2 (IP = 220 bar) and Blend-2 (IP = 240) are due to the presence of higher energy content caused by adding of H_2_ and the volatility nature of the fuel that makes a complete combustion. The maximum HRR for the fuel samples such as diesel, Blend-1, Blend-2 (IP = 180 bar), and Blend-2 (IP = 200 bar) are 56.5 J/°CA, 55.41 J/°CA, 55.63 J/°CA, and 60.98 J/°CA, which occurred at 11.5°CA, 10.3°CA, 10.9°CA, and 9.2°CA, respectively at full load.

### Performance characteristics

#### Brake-specific fuel consumption

**Fig. 8:**
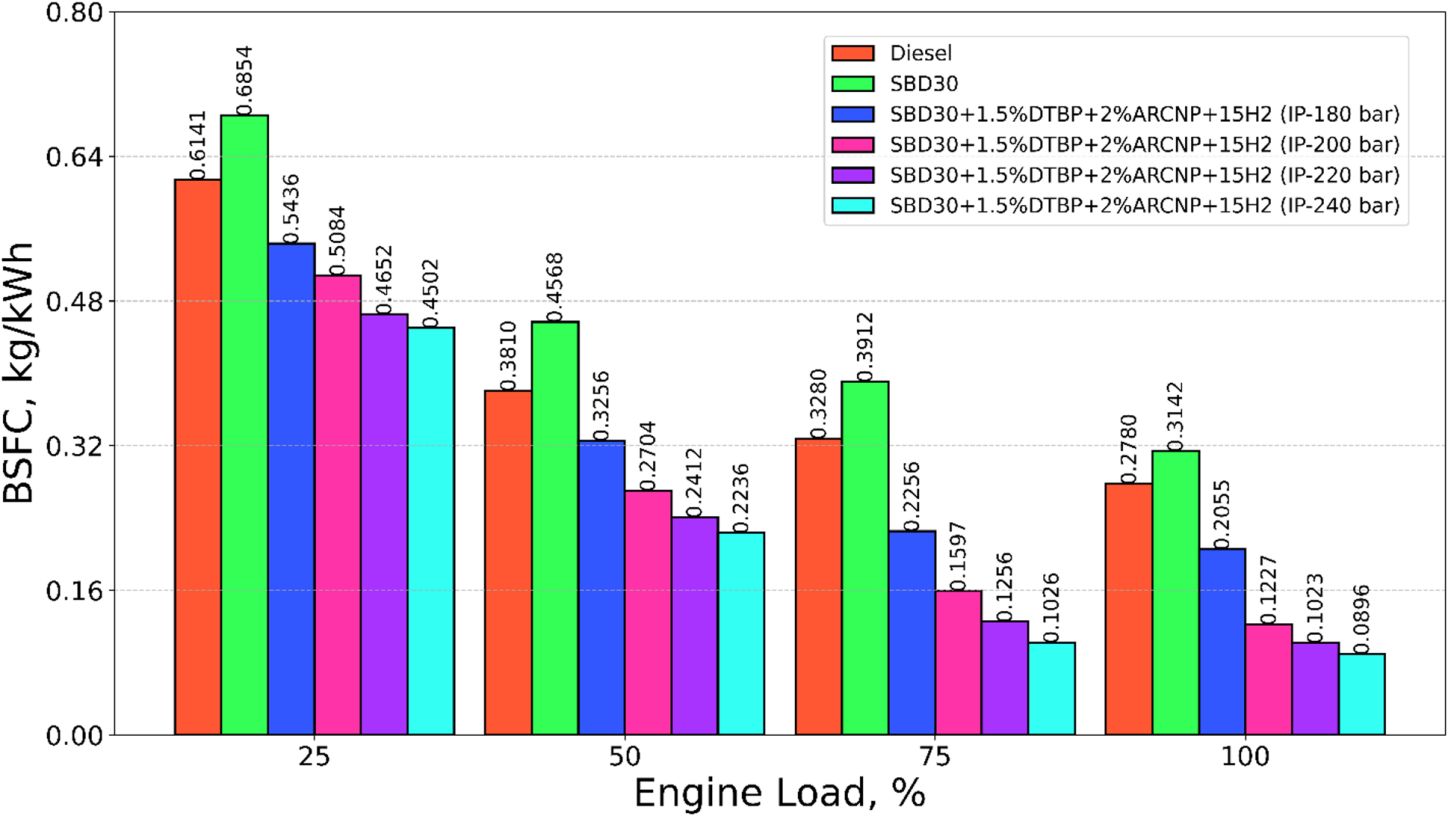
BSFC vs. engine load.

The brake-specific fuel consumption (BSFC) of the test fuels, diesel, Blend-1, Blend-2 with varying IP of 180, 200, 220, and 240 bar at different engine loadings is depicted in Fig. 8. The BSFC indicates the fuel quantity consumed by the engine to carry the load, keeping the speed of the engine constant. As it is observed, BSFC reduces on increasing the load. This phenomenon is attributed to high cylinder temperature. Among the fuels tested, Blend-1 offers the maximum BSFC. However, the Blend-2 (IP = 240 bar) gives the lowest BSFC. This is caused by rapid combustion of fuel with Cetane improver, availability of H_2_ molecules, as well as quality spray at 240 bar pressure, making good atomization and complete combustion of the fuel. The BSFC for Blend-2 (IP = 220 bar) is 12.4% higher than Blend-2 (IP = 240 bar) and 63.2%, 67.4%, 50.2%, and 16.6% lower than Diesel, Blend-2, Blend-2 (IP = 180 bar) Blend-2 (IP:200 bar), respectively at full load.

#### Brake thermal efficiency

**Fig. 9:**
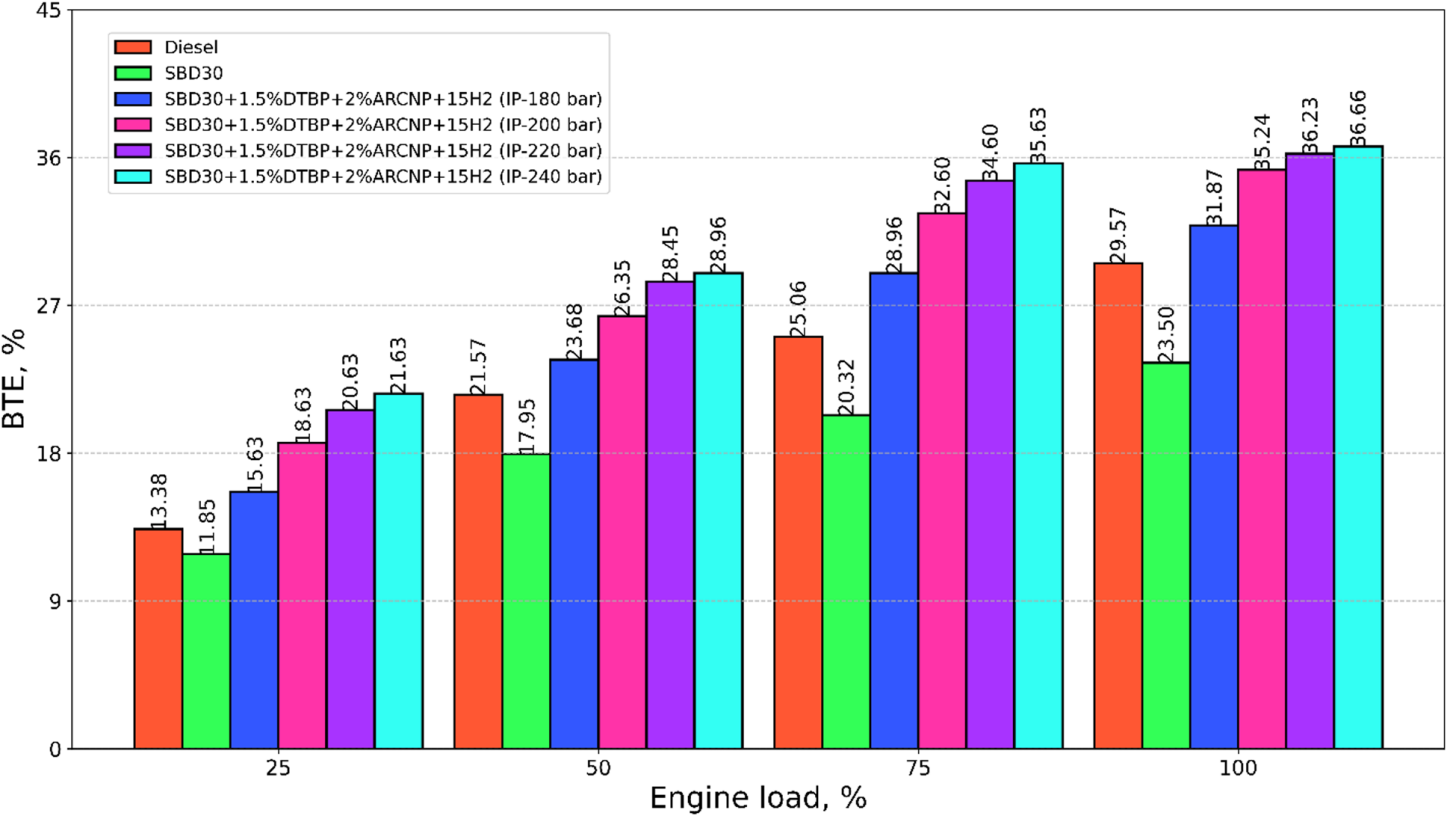
BTE vs. engine load.

The brake thermal efficiency **(**BTE) of the test fuels, such as diesel, Blend-1, Blend-2, with varying IP of 180, 200, 220, and 240 bar, is portrayed in Fig. 9. BTE indicates the energy conversion of the fuel in the form of useful work from the thermal energy generated during the combustion of the fuel. This is directly influenced by the CV of the fuel as well as the viscosity of the fuel. Blend-1 offers lower BTE compared to all the other test fuels, and this is due to the low energy content in Blend-1 compared to other fuels; however, for the combination, Blend-2 (IP:240 bar), the BTE is 36.7% at full load.

It is also observed that higher IP results in improved BTE, and this may be attributed to good spray and easy mix ability of the sprayed liquid fuel with the inducted H_2_. The combined effect may lead to increased BTE. The BTE for Blend-2 (IP = 220 bar) is 18.4%, 35.1%, 12.0%, and 2.7% higher than diesel, SBD30, Blend-2 (IP = 180 bar), Blend-2 (IP = 200 bar), respectively, at full load.

#### Exhaust gas temperature

**Fig. 10:**
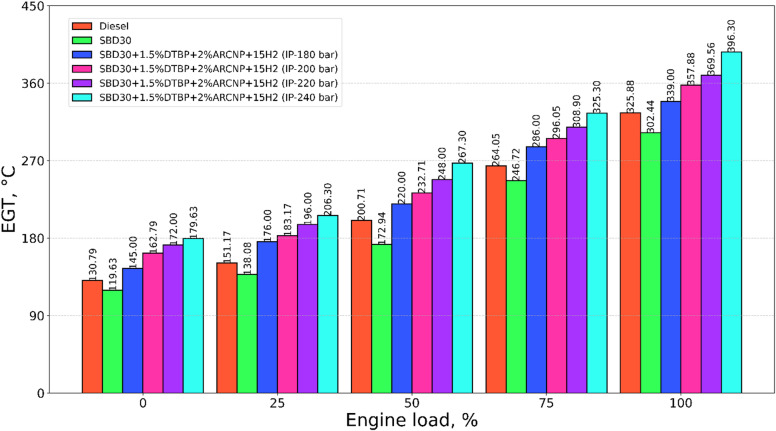
EGT vs. engine load.

The exhaust gas temperature (EGT) for diesel, Blend-1, Blend-2, of 180, 200, 220, and 240 bar is displayed in Fig. 10. The EGT in the case of fuel increases with a rise at higher loads owing to the high temperature of the cylinder at high loads. The peak GT in the case of diesel is 325.9 °C, which was higher by 7.2% than blend-1. However, Blend-2, combined with elevated IP show a rise in EGT. It is attributed to better combustion and higher combustion temperature during the combustion of H_2_ in the engine cylinder. The maximum EGT of 396.3 °C was observed for IP 240 bar at full load.

### Emission characteristics

#### CO emission

**Fig. 11:**
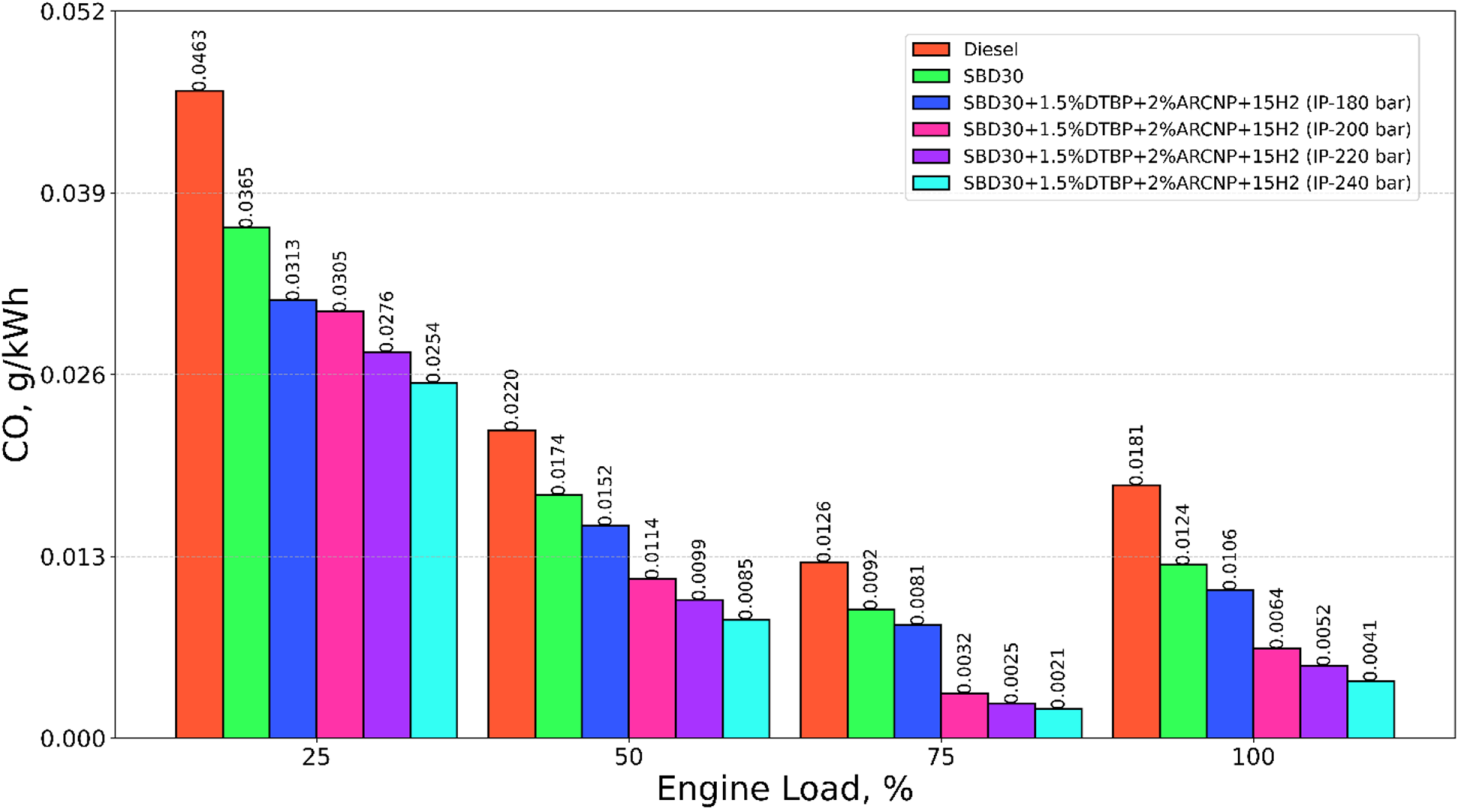
CO emission vs. engine load.

Figure 11 displays the variation of CO emission with varying engine loads for test fuels such as diesel, Blend-1, Blend-2, with varying IP of 180, 200, 220, and 240 bar. The CO emission for diesel is higher than all other fuels; this may be due to the existence of incomplete oxidation of the fuel due to the unavailability of oxygen in comparison to other fuel blends. The H_2_ – Blend-1 DF operation with DTBP and ARCNP at 240 bar IP releases the lowest CO emission compared to other combinations, owing to higher oxygen content in Blend-1 and DTBP and the formation of various ignition centers by ARCNP and clean combustion nature of H_2_ lead to lower CO emission as well as the high IP of 240 bar makes the pilot injected fuel into very fine droplets for easy mix ability and maintaining proper air-fuel ratio for complete combustion^65^. All these consequences collectively lead to a reduction in CO emissions. The CO emission for Blend-2 (IP = 220 bar) is 21.2% hgher than that of Blend-2 (IP = 240) and 71.1%, 57.7%, 50.5% and 17.8% lower than diesel, Blend-1, Blend-2 (IP = 180 bar), Blend-2 (IP = 200 bar) at full load, respectively.

#### HC emission

**Fig. 12:**
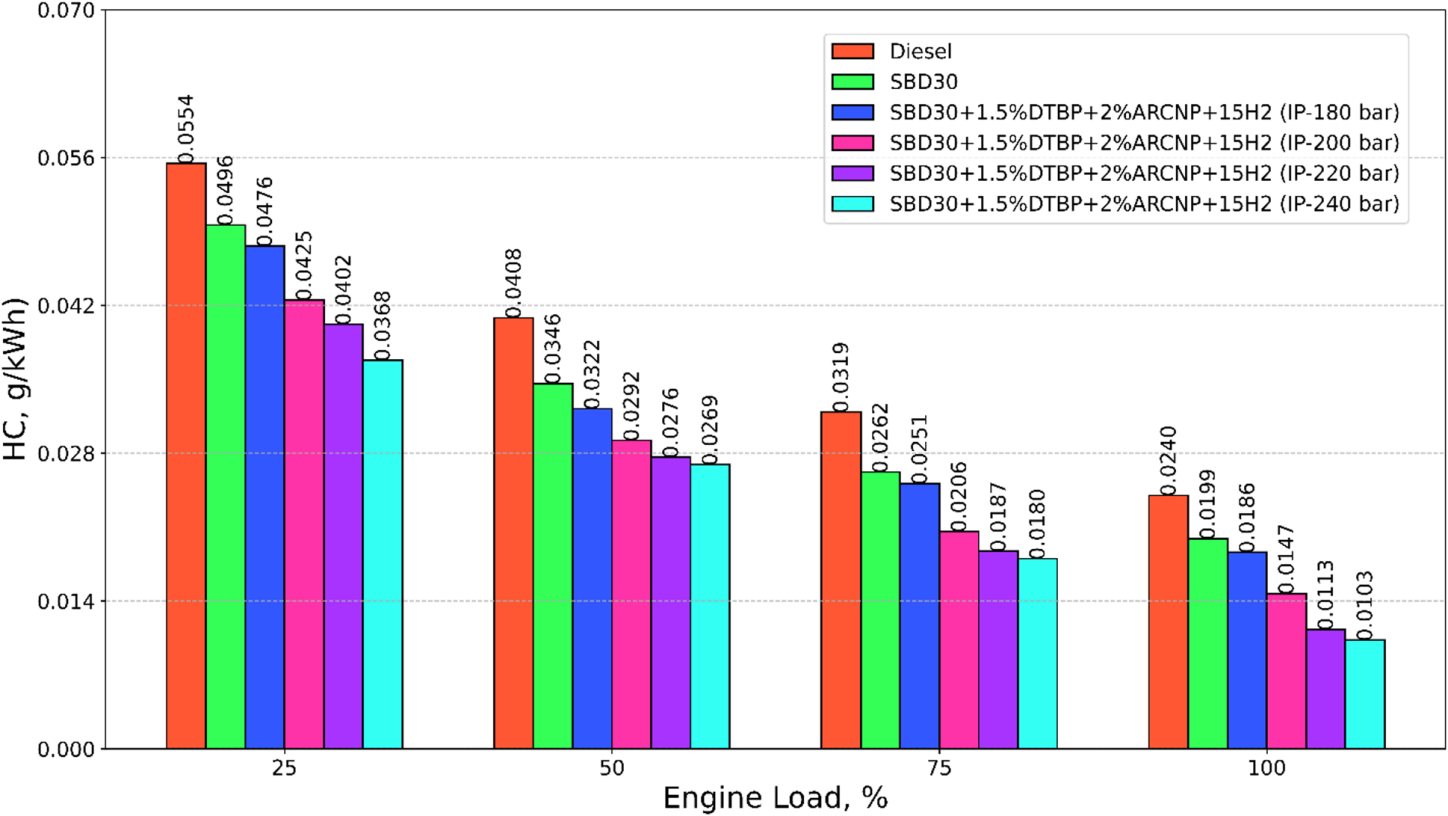
HC emission vs. engine load.

Figure 12 illustrates the variation of HC emission with varying loads for test fuels such as diesel, Blend-1, Blend-2 with varying IP of 180, 200, 220, and 240 bar. The HC emission reduces as the load increases for all the test fuels. This is attributed to a rise in ignition energy, better spray atomization, greater turbulence intensity, more heat transfer through the cylinder to the air-fuel mixer, and a rise in different ignition centres in the combustion chamber. The HC emission for the DF operation decreases gradually as IP increases. This could be due to superior H_2_ combustion and the presence of excess oxygen in Blend-1 and 1.5DTBP, which reduces the deficit in volumetric efficiency caused by H_2_ induction through the intake manifold. Additionally, the clean-burning characteristics of elemental H_2_ mitigate HC emissions. The HC emission for Blend-2 (IP = 220 bar) is 8.8% higher than that of Blend-2 (IP = 240) and 53.2%, 43.3%, 39.4% and 23.5% lower than diesel, Blend-1, Blend-2 (IP = 180 bar), Blend-2 (IP = 200 bar) at 100% engine load respectively.

#### NOx emission

**Fig. 13:**
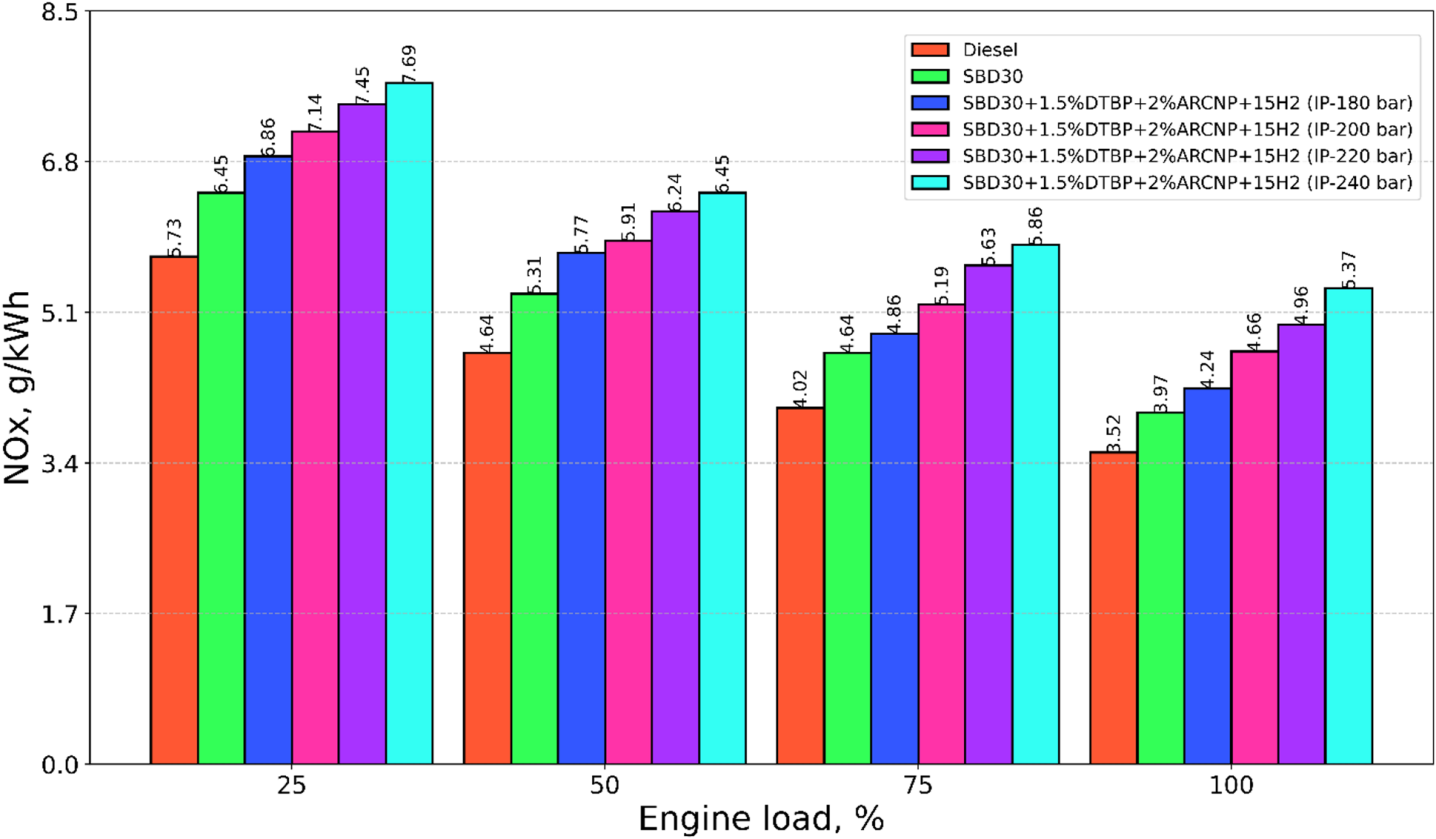
NOx emission vs. engine load.

The variation in NOx emission for investigated fuels, including diesel, Blend-1, Blend-2 with varying IP of 180, 200, 220, and 240 bar is shown in Fig. 13. NOx emission is susceptible to higher cylinder temperature, oxygen availability, engine CR, and time duration for the air-fuel mixture for combustion. For H_2_-DF operation with an increase in IP, the NOx increases drastically than that of Blend-1. This may be due to the high combustion temperature achieved during elevated IP with fire droplet size and combined effect of DTBP and Blend-1, which in turn lead to contributing high oxygen molecules, making more NOx emission. The NO emission for diesel is lower than all the fuels tested. This is because mineral diesel does not contain dissolved oxygen^67,68^. The specific NOx emission for Blend-2 (IP = 220 bar) is 8.1% lower than that of Blend-2 (IP = 240) and 40.9%, 25.0%, 17.2% and 6.5% higher than diesel, Blend-1, Blend-2 (IP = 180 bar), Blend-2 (IP = 200 bar) at full load, respectively.

#### Smoke emission

**Fig. 14:**
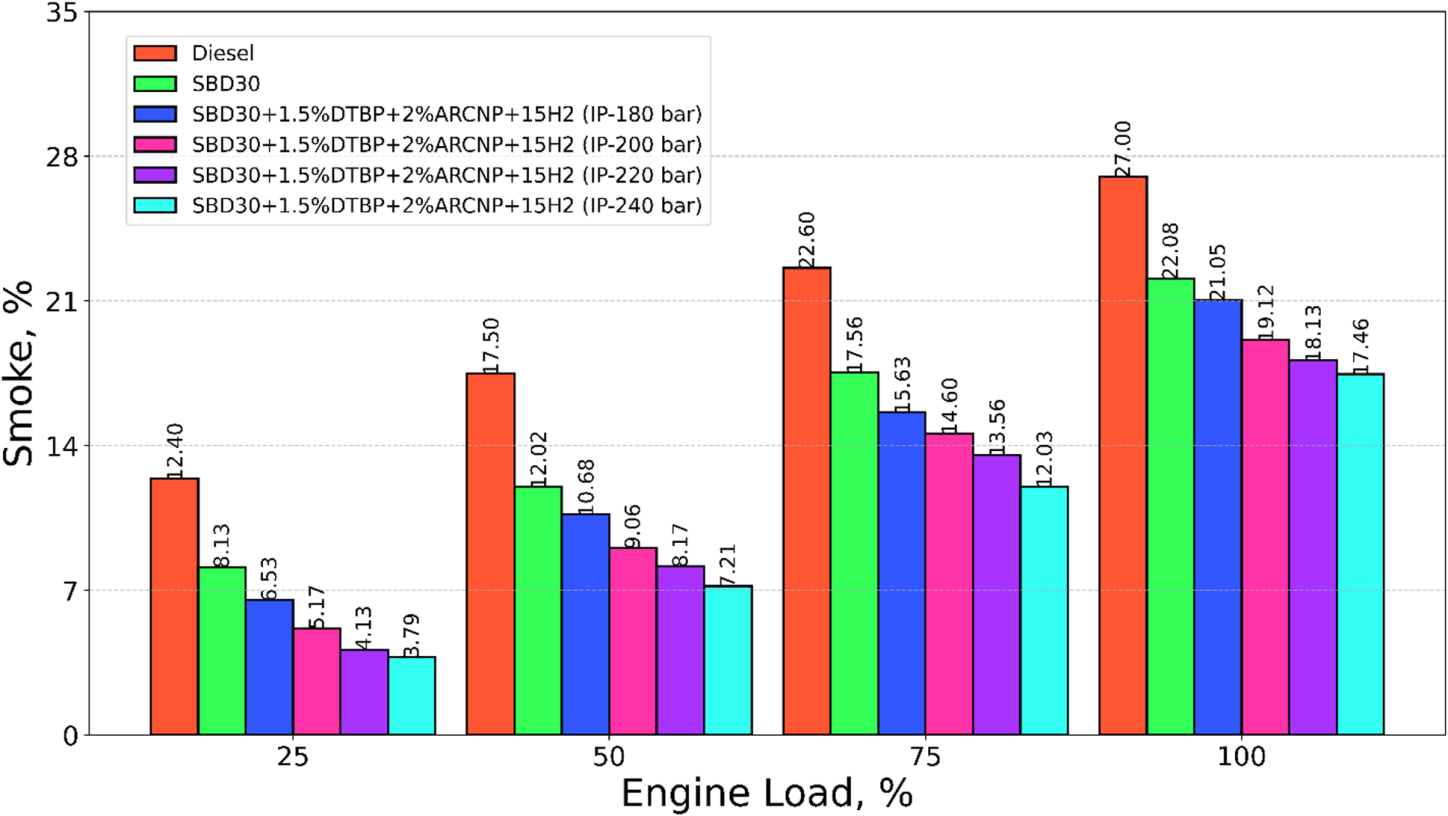
Smoke emission vs. Engine load.

The smoke emission for test fuels, including diesel, Blend-1, Blend-2 with varying IP of 180, 200, 220, and 240 bar, with varying load, is shown in Fig. 14. The smoke emission for diesel is extensively greater than Blend-1 and H_2_ DF operation with a change in IP. The high smoke in diesel is attributed to its aromatic compound’s availability in it. On the other hand, the smoke is reduced when H_2_ is injected since it has a little tendency to produce soot, and it does not include any components that are inferior to those found in the paraffin family’s composition. As an additional point of interest, the reduction in smoke may be linked to the increased oxidation of the soot particles in the soot-generating zone. This is because the number of hydrogen and oxygen molecules that are around the flame causes the particles to undergo a larger degree of oxidation, which ultimately results in a reduction in smoke emission^69,70^. The smoke emission for Blend-2 (IP = 220 bar) is 3.7% higher than that of Blend-2 (IP = 240) and 32.9%, 17.9%, 13.9% and 5.2% lower than diesel, Blend-2 (IP = 180 bar) Blend-2 (IP = 200 bar) at full load respectively.

## Machine learning model-prediction

### Data preprocessing

**Fig. 15:**
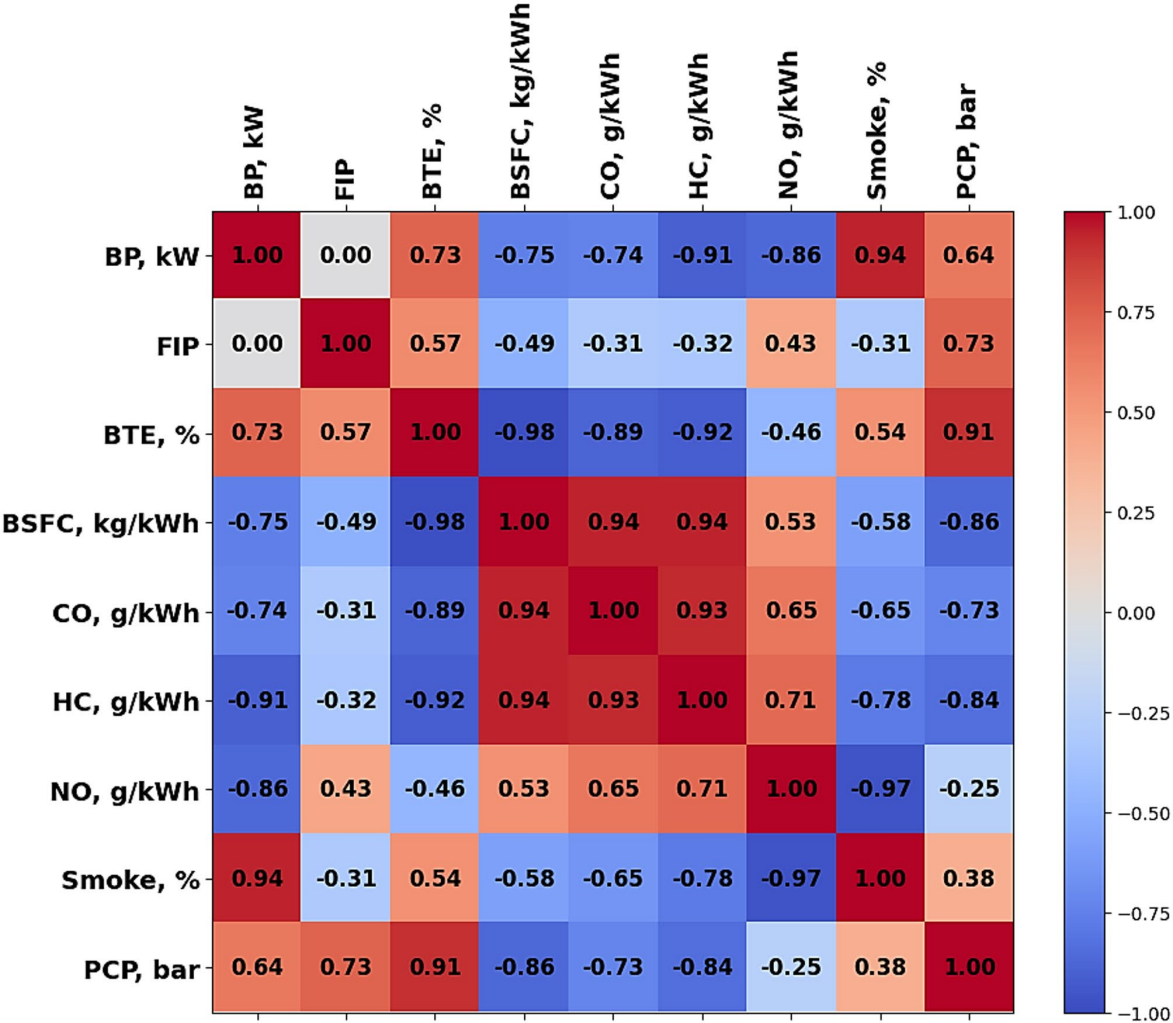
Correlation heatmap of engine combustion, performance, and emission parameters. [Heatmap was generated using Python 3.10 with Seaborn 0.12.2 and Matplotlib 3.7.1 (Seaborn documentation: https://seaborn.pydata.org; Matplotlib documentation: https://matplotlib.org)].

Quantifiable data on the correlations between engine performance and emission characteristics is provided by the correlation values (Table 4) between various data columns. BP and BTE have a significant positive correlation coefficient of 0.73, meaning that BTE increases by about 0.73 units for every unit increase in BP. By contrast, the strong negative correlation (-0.75) between BP and BSFC suggests that BSFC tends to decrease by around 0.75 units for every unit increase in BP. As BP rises by one unit, NO emissions fall by roughly 0.86 units, according to the strong negative correlation coefficient of -0.86 between the two variables. In a similar vein, the high positive relationship shown by the correlation coefficient of 0.94 between BP and smoke emission implies that the percentage of smokers increases by roughly 0.94 units for every unit increase in blood pressure. The precise numerical insights these correlation values offer into the mutual dependence of engine parameters enable the modification of engine performance and emission properties. Figure 15 depicts the correlation heatmap for the data employed in this study.

**Table 4:** Correlation values for data columns.

	BP, kW	FIP	BTE, %	BSFC, kg/kWh	CO, g/kWh	HC, g/kWh	NO, g/kWh	Smoke, %	PCP, bar
BP, kW	1.00	0	0.73	− 0.75	− 0.74	− 0.91	− 0.86	0.94	0.64
FIP	0.00	1.	0.57	− 0.49	− 0.31	− 0.32	0.43	− 0.31	0.73
BTE, %	0.73	0.57	1	− 0.98	− 0.89	− 0.92	− 0.46	0.54	0.91
BSFC, kg/kWh	− 0.75	− 0.49	− 0.98	1	0.94	0.94	0.53	− 0.58	− 0.86
CO, g/kWh	− 0.74	− 0.31	− 0.89	0.94	1	0.93	0.65	− 0.65	− 0.73
HC, g/kWh	− 0.91	− 0.32	− 0.92	0.94	0.93	1	0.71	− 0.78	− 0.84
NO, g/kWh	− 0.86	0.43	− 0.46	0.53	0.65	0.71	1	− 0.97	− 0.25
Smoke, %	0.94	− 0.31	0.54	− 0.58	− 0.65	− 0.78	− − 0.97	1	0.38
PCP, bar	0.64	0.73	0.91	− 0.86	− 0.73	− 0.84	− 0.25	0.38	1

The performance and emissions aspects of the engine are adequately depicted by the descriptive statistics, as listed in Table 5. BP is the most notable of these, averaging 2.08 kW and showing a little variation around this mean value, as seen by a 1.06 KW standard deviation. By contrast, FIP has a mean of 220 and a standard variation of 29. With a standard deviation of 7.56%, the BTE averages 26.46% and is somewhat variable. With a mean value of 0.3 kg/kWh and a standard deviation of 0.172 kg/kWh, BSFC on the other hand, exhibits steady trends. Similar emissions values of 0.014 g/kWh for CO and 0.028 g/kWh for HC show significant variability with standard deviations of 0.011 g/kWh and 0.012 g/kWh, respectively. The standard deviation was 5.732 g/kWh in NOx emissions. There is reasonable inconsistency specified by the smoke percentage of about 12.31% and the standard deviation of 5.71%. The mean of 72.85 bar and standard deviation of 6.4 bar describe the diffident inconsistency of PCP. Further details concerning the distributional features of the data are given by skewness and kurtosis values, which also showcase the symmetry and peakiness of the distributions across parameters. These comprehensive statistics serve as the cornerstone for further research and optimization initiatives by offering vital information on the engine’s operating efficiency and emission characteristics.

**Table 5:** Descriptive statistics.

	BP, kW	FIP	BTE, %	BSFC, kg/kWh	CO, g/kWh	HC, g/kWh	NO, g/kWh	Smoke, %	PCP, bar
Count	20	20	20	20	20	20	20	20	20
Mean	2.08	220	26.46	0.3	0.014	0.028	5.732	12.31	72.85
std	1.06	29	7.56	0.172	0.011	0.012	1.052	5.71	6.4
min	0.8	180	11.85	0.09	0.002	0.01	3.97	3.79	61.8
25%	1.393	200	20.55	0.151	0.006	0.019	4.938	7.9	68.4
50%	1.95	220	27.4	0.256	0.01	0.027	5.698	12.02	73.17
75%	2.63	240	33.1	0.452	0.019	0.035	6.451	17.48	77.03
max	3.62	260	36.66	0.685	0.037	0.05	7.685	22.08	83.9
Skewness	0.35	0	− 0.23	0.598	0.907	0.417	0.247	0.13	− 0.09
Kurtosis	− 1.18	− 1.3	− 1.08	− 0.519	− 0.426	− 0.684	− 0.712	− 1.2	− 0.76

### Engine combustion and performance models

Three primary engine combustion and performance characteristics, namely BTE, BSFC, and PCP, were employed for model development of LR, DTs, and RF.

**Fig. 16:**
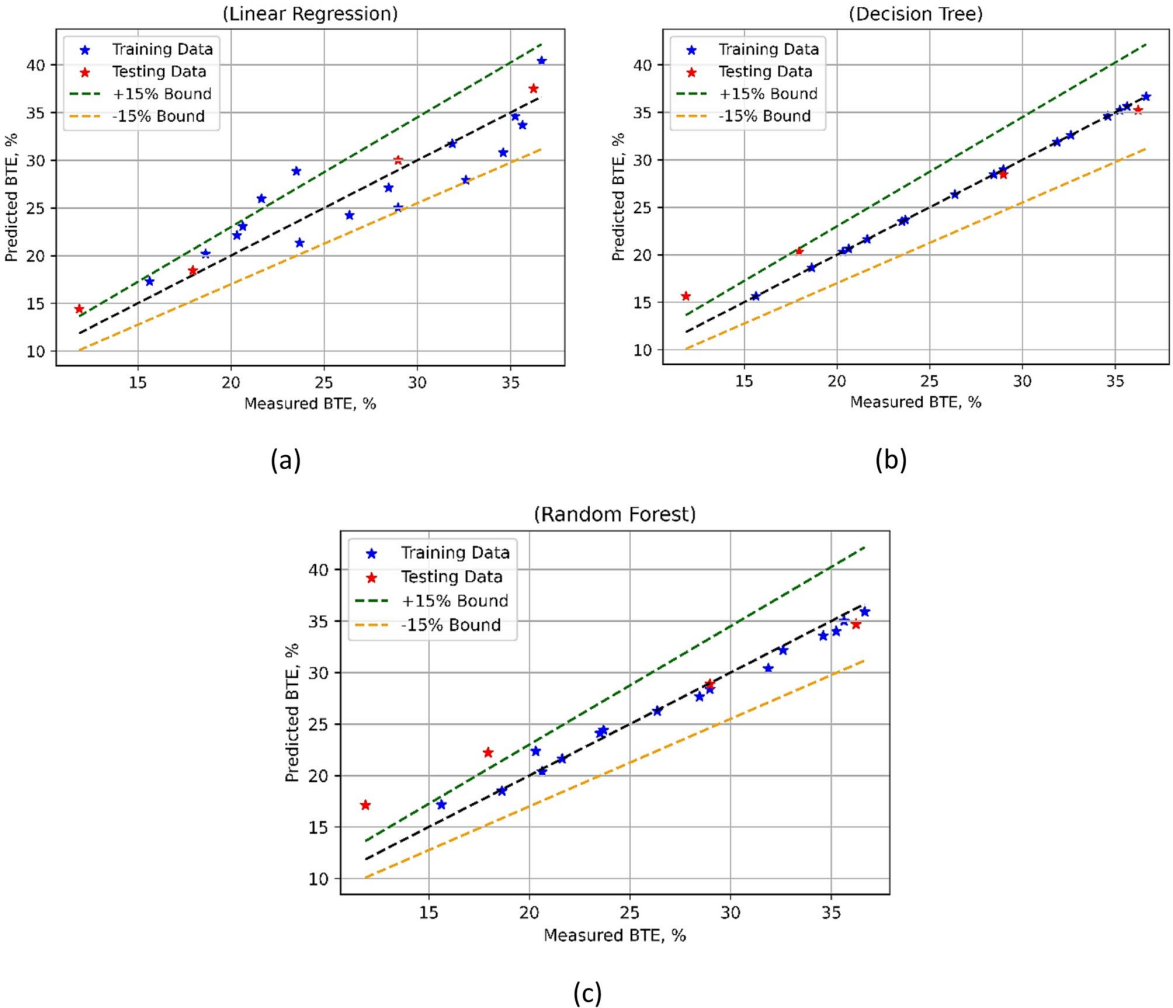
BTE model’s measured vs. model prediction values for (**a**) LR; (**b**) DT, and (**c**) RF ML techniques.

The model performance in the case of BTE is compared in Fig. 16. An extensive assessment of the predictive power of different algorithms is given by the model performance indicators, as listed in Table 6.

The test MSE of Linear Regression (LR) is much lower at 2.3807 than the training MSE of 8.9671. Strong predictive performance is shown by the R-squared (R^2^) values; the test R^2^ is substantially higher at 0.9734 than the training R^2^ of 0.7924. Low Willmott’s Index (Wi) values for training (0.9396) and testing (0.9932) of LR also suggest that there are few mistakes in target variable prediction. With the training MAPE of 10.1182% and the test MAPE of 7.8782%, the Mean Absolute Percentage Error (MAPE) for LR is, nevertheless, noticeably larger. Decision Trees (DT) on the other hand, achieve a higher test MSE of 5.2821 but a training MSE of 0, which denotes a perfect fit. Predictive abilities are excellent as seen by the high R2 values for both training (1.0000) and testing (0.9410). DT’s larger Wi values for testing (0.9818) and training (0.9998I shows good predictive efficiency. With a test MSE of 12.1378, and a training MSE of 0.9027, Random Forests (RF) show a balance between bias and variance. Strong R^2^ scores (0.9791) and (0.8644) for training and testing, respectively, suggest good predictive performance. For training (0.9942) and testing (0.9539), RF likewise keeps low Wi values, suggesting modest prediction errors. With a training MAPE of 3.0541% and a test MAPE of 18.2396%, RF does, nevertheless, show higher MAPE values than LR. It was noted that the BTE models developed with DT was superior to the other two approaches as depicted in Fig. 16, that DT based BTE model was the best performing model. Through the provision of information about the advantages and disadvantages of each model, these indicators facilitate better decision-making in assignments involving predictive modelling.

**Table 6:** Statistical results of developed models.

Parameter	Model	Train MSE	Test MSE	Train R2	Test R2	Train Wi	Test Wi	Train MAPE	Test MAPE
BTE	LR	8.9671	2.3807	0.7924	0.9734	0.9396	0.9932	10.12	7.8782
	DT	0	5.2821	1.0000	0.9410	0.9998	0.9818	0	12.3939
	RF	0.9027	12.1378	0.9791	0.8644	0.9942	0.9539	3.05	18.2396
BSFC	LR	0.0056	0.0057	0.7374	0.8870	0.9199	0.9661	33.19	18.1883
	DT	0.0000	0.0063	1.0000	0.8744	1.0000	0.9574	0	15.7166
	RF	0.0002	0.0088	0.9884	0.8233	0.9970	0.9390	5.761	19.0762
PCP	LR	2.6882	0.4655	0.9126	0.9932	0.9766	0.9984	2.01	0.8649
	DT	0.0000	1.4328	1.0000	0.9792	1.0000	0.9942	0	1.4213
	RF	0.9623	4.1308	0.9687	0.9401	0.9908	0.9808	1.09	2.8479

**Fig. 17:**
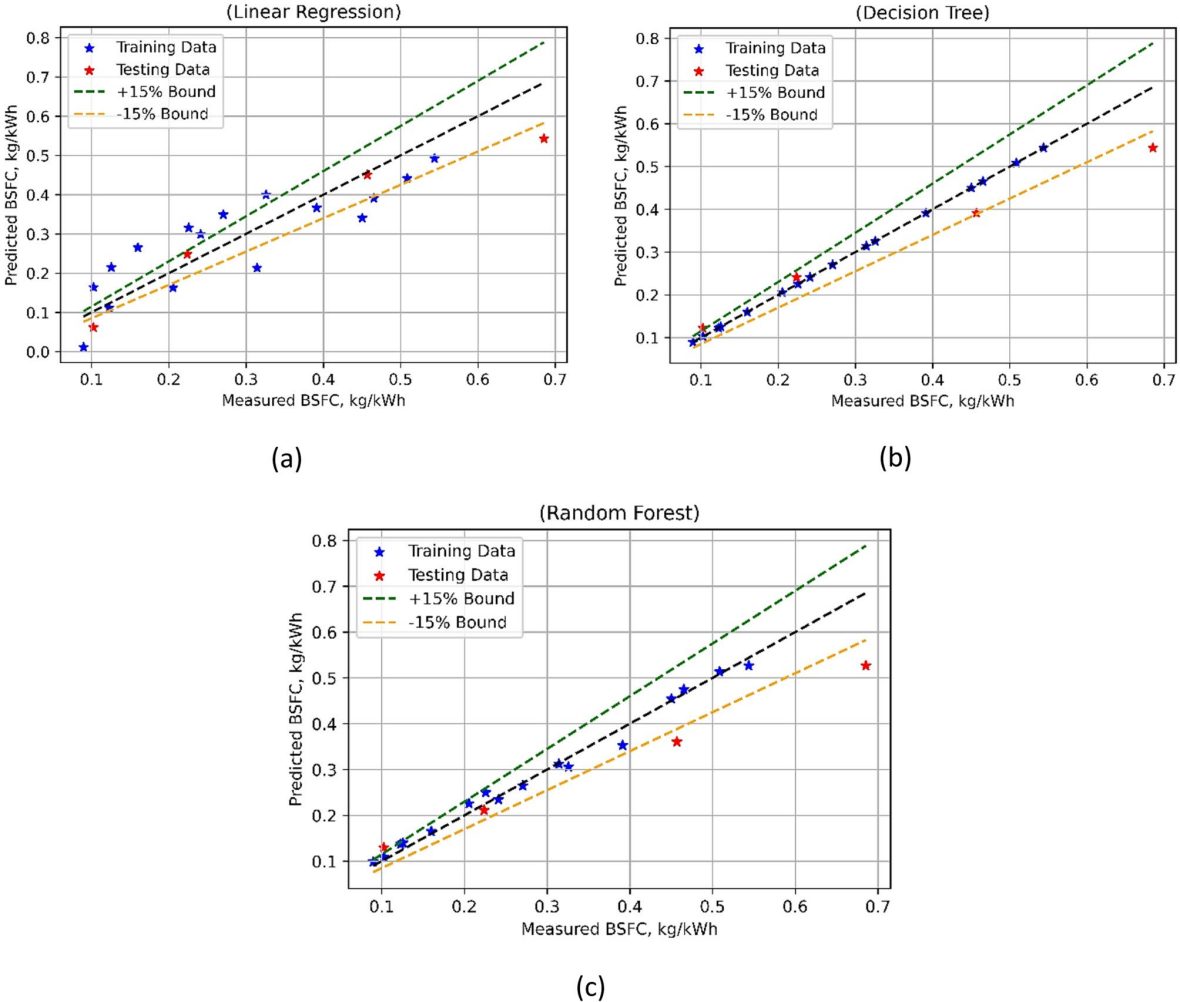
BSFC model’s measured vs. model prediction values for (**a**) LR; (**b**) DT and (**c**) RF ML techniques.

**Fig. 18:**
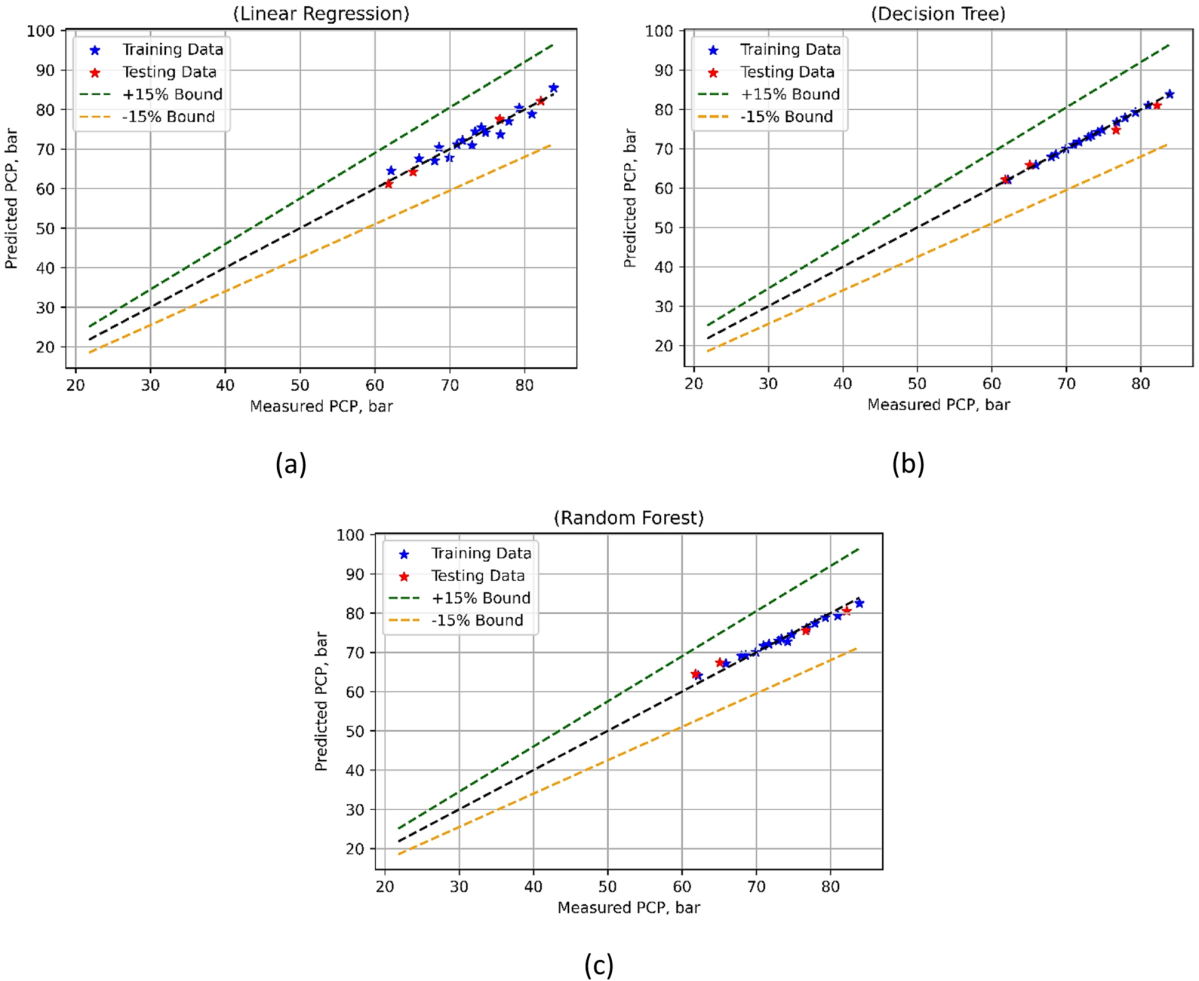
PCP model’s measured vs. model prediction values for (**a**) LR; (**b**) DT and (**c**) RF ML techniques.

Once the BSFC models were developed these were used predictions. A comparative graph for the three ML techniques used for BSFC is shown in the Fig. 18. A detailed evaluation of the forecasting capacities of all three ML approaches in the case of BSFC models is provided by the model performance metrics listed in Table 6. A slightly higher test MSE as 0.0057 and a lower MSE (training) as 0.0056 characterize linear regression L). Results for R^2^ range from moderate to excellent predictive performance; the test R^2^ is 0.8870 and the training R^2^ is 0.7374. LR also has rather low Willmott’s Index (Wi) values for testing (0.9661) and training (0.9199), showing strong agreement between predicted and observed values. With MAPE (training) of 33.1917% and MAPE (test) of 18.1883%, the MAPE for LR is, however, much higher.

DT on the other hand has a higher MSE (during testing phase) of 0.0063 but a training MSE of 0, depicting a robust match. High R^2^ scores for testing (0.8744) and training (1.0000) continue to suggest very good prediction abilities. At training, MSE of 0.0002 and test MSE of 0.0088, Random Forests (RF) achieve a balance of bias and variance. Strong R^2^ scores (0.9884) and (0.8233) for testing and training respectively suggest good predictive performance. Good agreement between predicted and observed values is also shown by RF’s low Wi values for training (0.9970) and testing (0.9390). But RF shows higher MAPE values than LR—a test MAPE of 19.0762% and a training MAPE of 5.7609%. Figure 17 shows that the DT-based BSFC model was the best-performing model. Combining these indicators sheds light on the advantages and disadvantages of each model, which facilitates better decision-making in BSFC predictive modeling projects. The prediction evaluations of Peak Cylinder Pressure (PCP) models using LR, DT, and RF are evaluated using the model performance metrics, listed in Table 6. The comparison is shown in Fig. 18. Whereas the test MSE for LR is 0.4655, the training MSE is 2.6882. With the test R^2^ much higher at 0.9932 and the training R2 at 0.9126, the R^2^ data indicate excellent predictive ability. Low Willmott’s Index (Wi) scores for training (0.9766) and testing (0.9984) further suggest that LR makes a few mistakes in target variable prediction. With MAPE (training) of 2.0061% but MAPE (testing phase) of 0.8649%, LR does, nevertheless, have a comparatively low MAPE. DT on the other hand has a higher test MSE of 1.4328 but a training MSE of 0, indicating a perfect match. High R^2^ scores for testing (0.9792) and training (1) continue to suggest very good prediction abilities.

With training MSE of 0.9623 and test MSE of 4.1308 RF exhibit a balance of bias and variance. Strong R^2^ scores (0.9687) and (0.9401) for testing and training respectively suggest good predictive performance. Wi values for training (0.9908) and testing (0.9808) are similarly kept rather low by RF, suggesting a respectable agreement between predicted and observed values. At MAPE (training) of 1.0854% while the MAPE in testing phase was 2.8479%, RF does, however, show higher MAPE values than LR. It is clear from Fig. 18 that the DT-based PCP model displayed the highest level of performance possible.

### Engine emission models

Three different engine emission models, namely CO, HC, NOx, and smoke emission, were developed using LR, DTs, and RF. The predictive performance of three ML-based prediction methods was used for developing carbon monoxide (CO) models, as listed in Table 7. These models were thoroughly investigated using the statistics-based model assessment metrics. With training and test MSE values almost zero, it showed accurate predictions even in the case of LR. A training R^2^ of 0.5862 and a test R^2^ of 0.7316 demonstrate that LR generates moderate to strong predictive precision. Training and testing Willmott’s Index (Wi) values of 0.8536 and 0.9119, respectively, suggest moderate agreement for LR. But LR has rather high MAPE values—96.9317% during training phase and 49.09% in the testing phase. For DT-based models, the MSE values were negligible both in the case of training as well as test sets, showing a robust model prediction. Similarly, strong predictive abilities are demonstrated by the R^2^ values for DT that stay high across the training (0.9998) and test (0.9397) datasets, and also in the case of higher Wi values—0.99998 for training and 0.9811 for testing.

**Table 7:** Statistical results of developed emission models.

Parameter	Model	Train MSE	Test MSE	Train *R*^2^	Test *R* ^2^	Train Wi	Test Wi	Train MAPE	Test MAPE
CO	LR	0	0	0.5862	0.7316	0.8536	0.9119	96.9317	49.0862
	DT	0	0	0.9998	0.9397	0.9998	0.9811	0	16.0871
	RF	0	0	0.9919	0.9026	0.9979	0.9697	11.6527	13.1490
HC	LR	0	0	0.9050	0.9579	0.9745	0.9887	13.1399	7.3132
	DT	0	0	0.9998	0.9710	1.0000	0.9915	0.0000	11.0758
	RF	0	0	0.9895	0.9294	0.9972	0.9771	3.7330	15.4095
NOx	LR	0.0718	0.0748	0.9402	0.8337	0.9844	0.9633	4.6421	4.5639
	DT	0	0.1314	0.9998	0.7079	0.9997	0.9368	0	6.1621
	RF	0.0109	0.0705	0.9909	0.8433	0.9976	0.9607	1.7032	3.6140
Smoke	LR	0.6708	0.2578	0.9803	0.9860	0.9950	0.9967	6.9747	3.0731
	DT	0	1.5605	1.0000	0.9155	0.9998	0.9812	0	12.3875
	RF	0.2283	1.7542	0.9933	0.9050	0.9983	0.9780	4.6818	13.0993

**Fig. 19:**
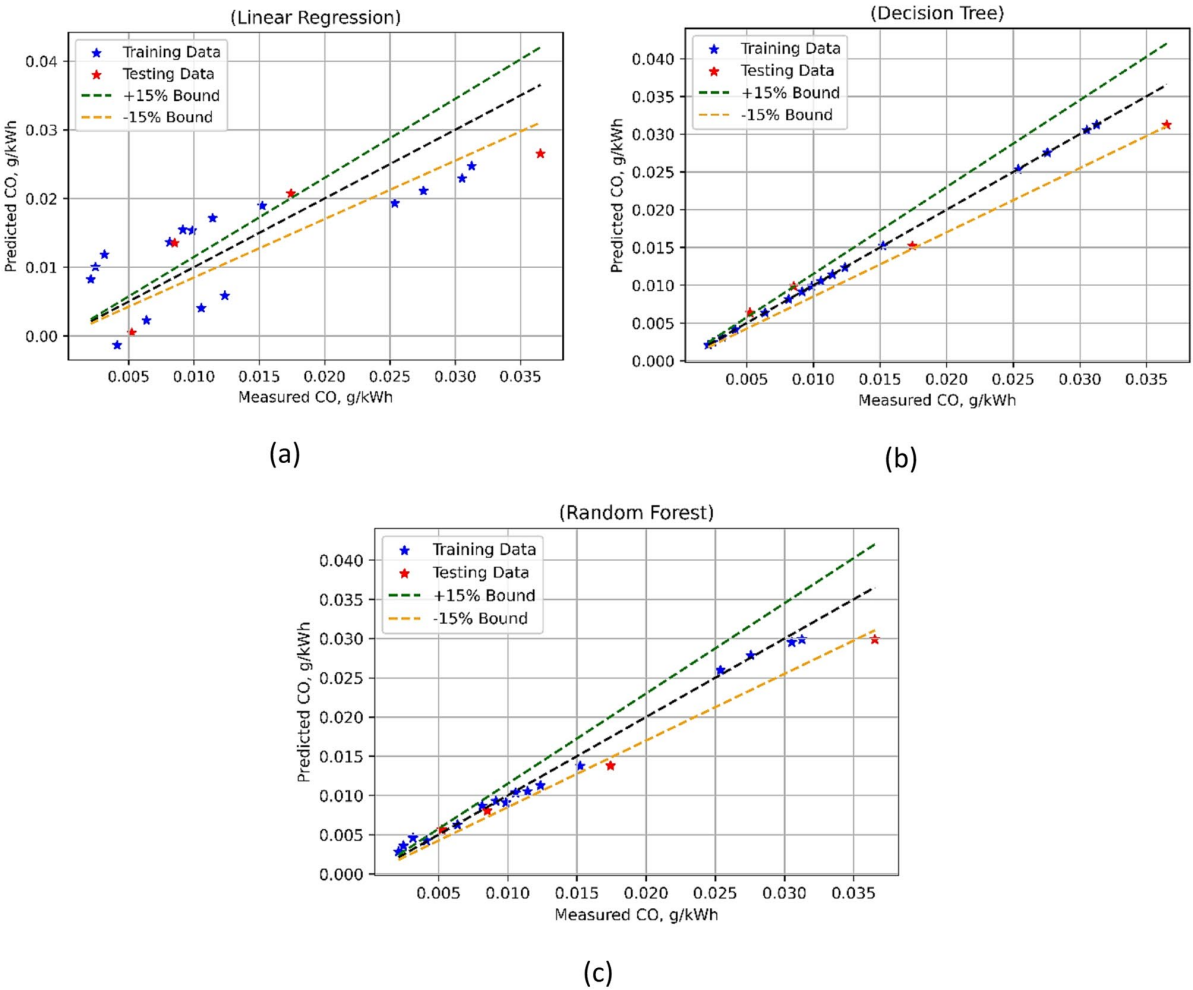
CO model’s measured vs. model prediction values for (**a**) LR; (**b**) DT, and (**c**) RF ML techniques.

With MSE values of 0 for both the training and test datasets, Random Forests (RF) also demonstrate an exact fit. The R² values, recorded as 0.9919 during training and 0.9026 for testing, indicate that RF provides a fairly strong predictive capability. Wi’s results confirm that the alignment between actual and predicted values remains satisfactory, with training and testing values of 0.9979 and 0.9697, respectively, falling within an acceptable range. However, RF produces relatively lower MAPE values, amounting to 11.6527% for training and 13.1490% for testing. Based on this analysis, it can be inferred that DT-based models outperformed the other two ML models. This conclusion is further reinforced by Fig. 19, where the DT-based CO emission model is observed to deliver the best performance.

The ML-based HC emission model’s predictive performance was evaluated using different statistical metrics, as listed in Table 7. A good performance was shown by the training as well as model testing MSE values for LR. Having a robust training R^2^ at 0.9050 and the test R^2^ at 0.9579, LR exhibits moderate to strong prediction accuracy. Willmott’s Index (Wi) scores of 0.9745 for training and 0.9887 for testing further demonstrate satisfactory agreement in LR. But for LR, the MAPE is rather low—13.14% for training and 7.32% for testing. The DT-based HC emission model exhibited a flawless match with negligible MSE values for both training and testing. Significant prediction abilities are shown by the R^2^ values for DT that are high for both the training (1.0000) and test datasets (0.9710).

**Fig. 20:**
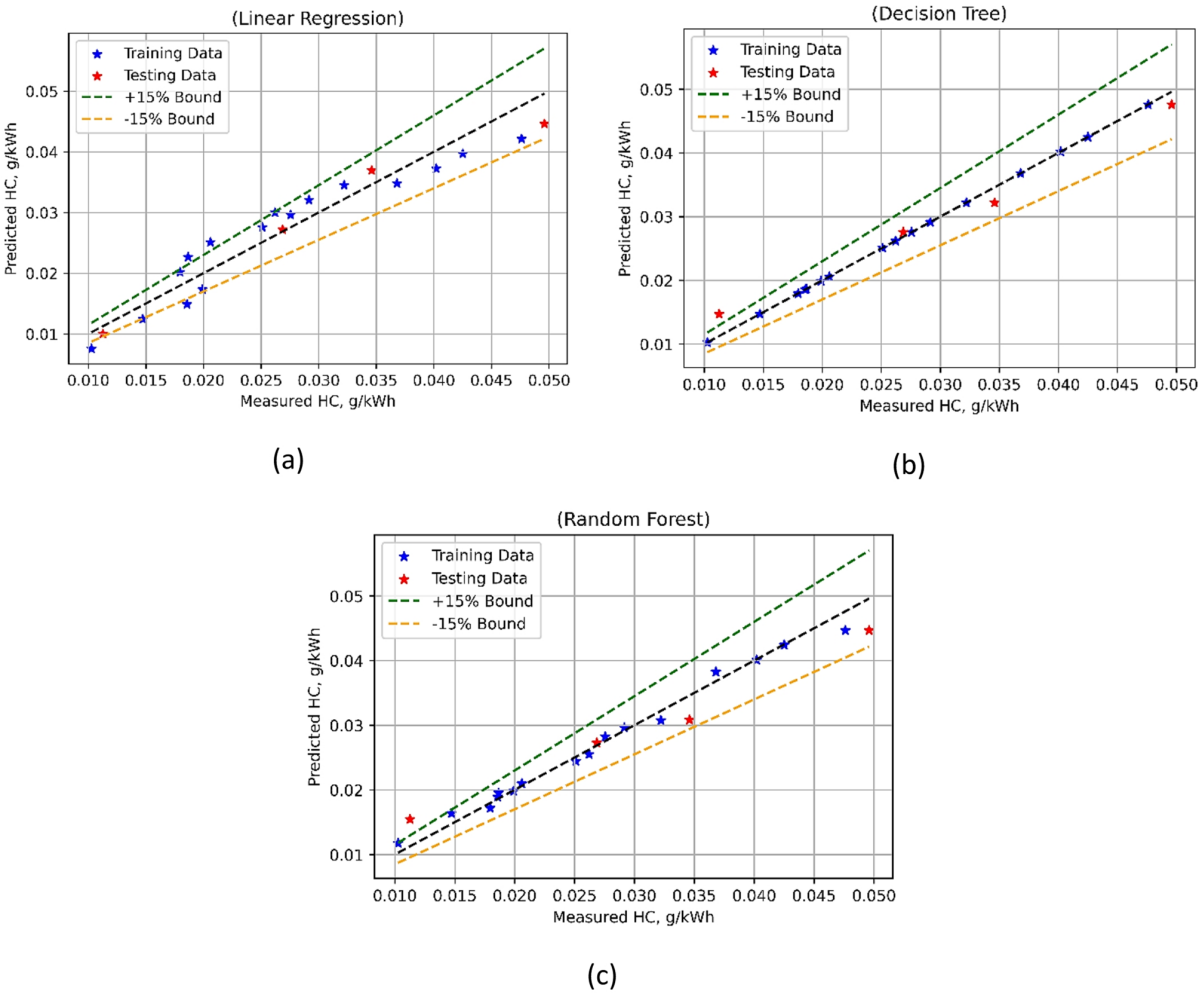
HC model’s measured vs. model prediction values for (**a**) LR; (**b**) DT, and (**c**) RF ML techniques.

Furthermore, the RF-based HC emission model also showed robust performance with almost negligible MSE values for the test and training sets. R^2^ (0.9895) during training and (0.9294) during the test show that RF performs reasonably well predictively. Nevertheless, RF yields quite low MAPE values compared to LR—3.7330% for training and 15.4095% for testing. It can be concluded using results in Table 7; Fig. 20, that DT-based HC emission models were superior to the other two. By fully describing the advantages and disadvantages of each model, these indicators facilitate well-informed choices in HC emission prediction modeling. The predicted performance of multiple algorithms-based NOx emissions model was comprehensively evaluated using the evaluation metrics, and outcomes are presented in the Table 7. For LR model, the results were pretty fair as indicated by the low training as well as testing MSE values of 0.0718 and 0.0748. The R^2^ values were 0.9402 during training phase and 0.8337 in testing phase, thus LR has a good prediction accuracy. Willmott’s Index (Wi) of 0.9844 for training and 0.9633 for test further indicates that LR exhibits satisfactory agreement. But at 4.6421% for training and 4.5639% for testing, the MAPE for LR is rather poor. The DT-based NOx models performed in a superior way. It showed a MSE (test phase) of 0.1314, while it was 0 for the model training phase test. For training, the R^2^ values for DT were high (0.9998), suggesting excellent predictive performance; for testing, they are much lower (0.7079).

**Fig. 21:**
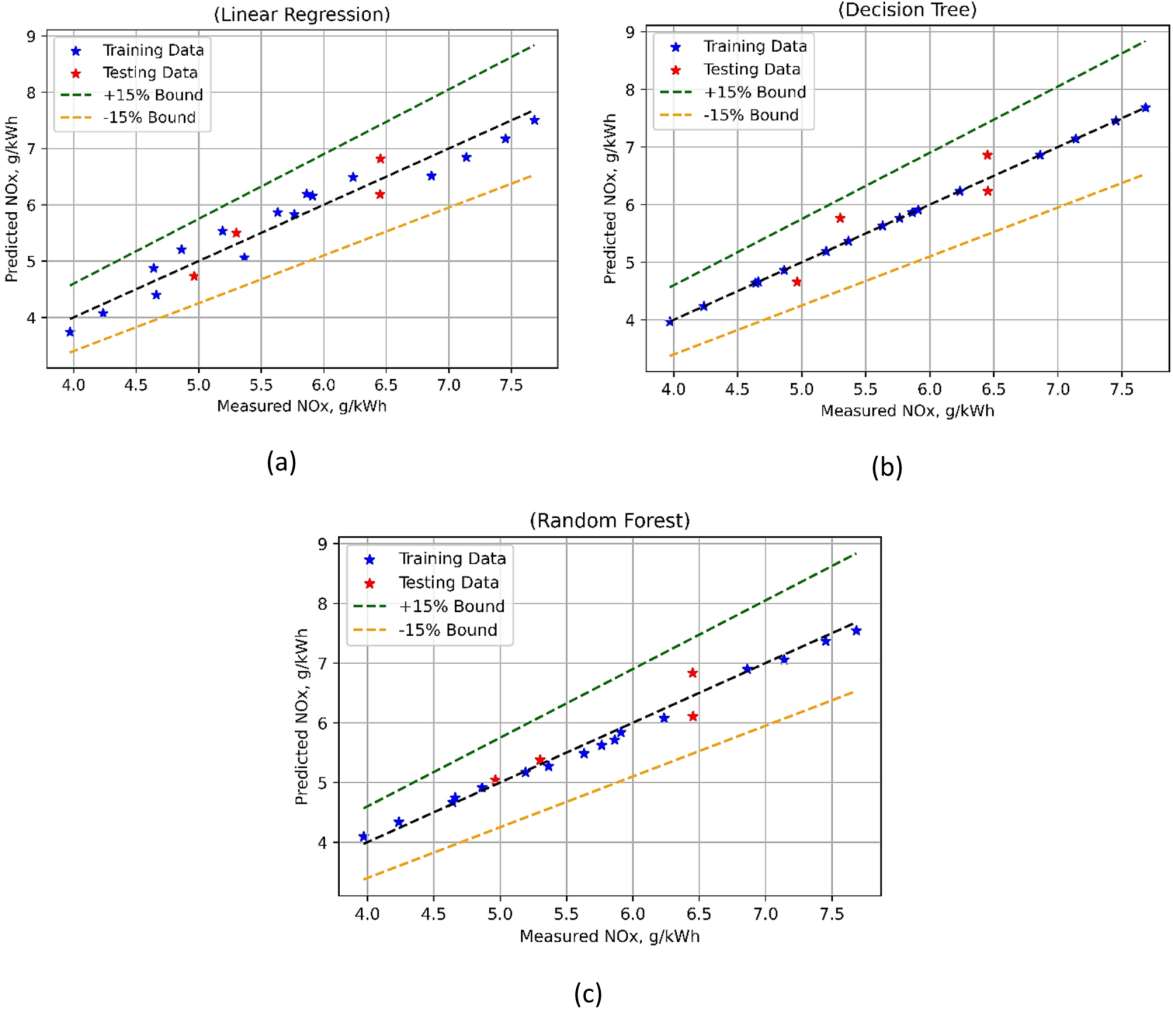
NOx model’s measured vs. model prediction values for (**a**) LR; (**b**) DT and (**c**) RF ML techniques.

At a test MSE of 0.0705 and a training MSE of 0.0109, Random Forests (RF) show balanced performance. R^2^ as 0.9909 for training and 0.8433 during model test indicate that RF performs very well predictively. At Wi values of 0.9976 for training and 0.9607 for testing, the agreement between predicted and observed values in RF is still good. Moreover, RF generates MAPE values that are rather low (1.7032% for training and 3.6140% for testing). Overall, it can be observed that the DT-based NOx emission model outperformed the other test methods as estimated using statistical evaluation (Table 7), as well as pictorial comparison (Fig. 21).

The smoke emissions model was developed employing three ML approaches was evaluated with a battery of statistical evaluation, as shown in Table 7. The models were also compared for their actual vs. model-predicted values. Evaluation criteria provide a detailed examination of the accuracy with which many algorithms forecast smoke emissions. The low training and test MSE values of 0.6708 and 0.2578, correspondingly, indicate a strong match starting with linear regression (LR).

**Fig. 22:**
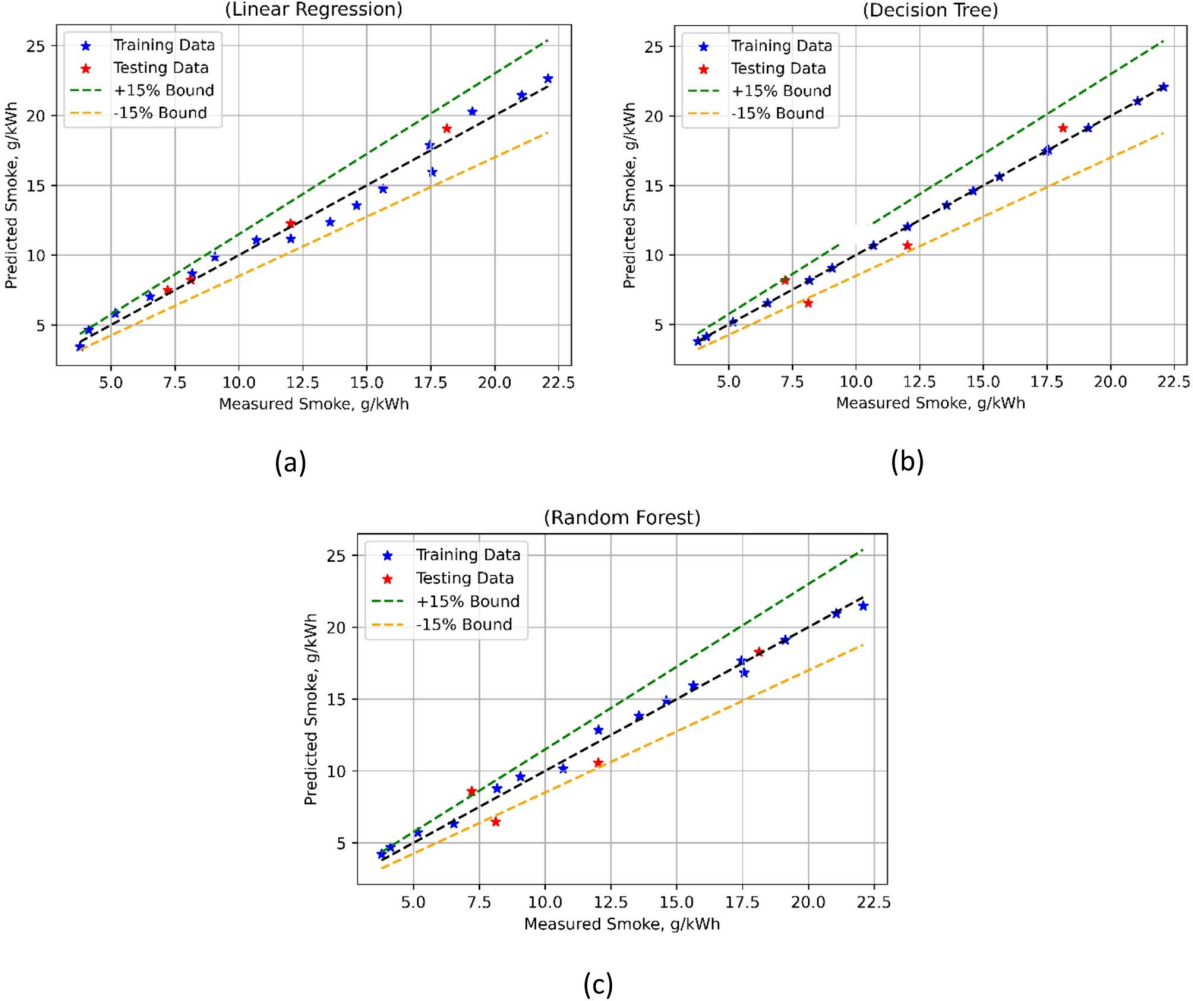
Smoke model’s measured vs. model prediction values for (**a**) LR; (**b**) DT, and (**c**) RF ML techniques.

High prediction accuracy of LR is achieved at training R^2^ of 0.9803 and test R^2^ of 0.9860. Willmott’s Index (Wi) values for model training and model test, as 0.995 and 0.9967, respectively, indicate that LR also shows good agreement. Still, the MAPE for LR is moderate at 6.9747% for training and 3.0731% for testing. DT also offers a respectable match with a test MSE of 1.5605 and almost negligible for training. The R^2^ values for DT have good predictive performance; however, they are much lower for testing (0.9155) than for training (0.9998). DT gets allowable agreement at Wi values of 1.0000 for training and 0.9812 for testing.

Results from Fig. 22 shows that the RF-based ML were balanced; their training MSE is 0.2283, and their test MSE is 1.7542. RF has a very good prediction performance with R^2^ as 0.9933 during training and 0.9050 for the model test. Relatively good agreement between predicted and observed values in RF is still shown by Wi values of 0.9983 during training and 0.9780 during the model test. Furthermore, RF generates MAPE values that are rather low (4.6818% for training and 13.0993% for testing). In this regard, too, the DT-based NOx simulation outperformed the other two techniques.

### Energy and life-cycle efficiency analysis

The integration of hydrogen and Spirogyra biodiesel as a dual-fuel combination offers a sustainable and energy-efficient alternative to conventional diesel. The Spirogyra-derived biodiesel, produced from renewable algal biomass, contributes to a carbon-neutral cycle by utilizing atmospheric CO₂ during growth and releasing it upon combustion. The presence of hydrogen, a clean subordinate fuel, further improves the combustion process by endorsing complete oxidation, increasing the BTE, and decreasing BSFC, mainly at optimized injection pressures of 220–240 bar. From an energy viewpoint, the DF mode attained higher energy conversion efficiency due to enhanced atomization and quicker flame propagation, while also minimalizing heat losses and unburned HC emissions. Life-cycle wise, when Spirogyra biodiesel is coupled with green hydrogen produced from renewable electrolysis, the combined system can suggestively lower the overall carbon footprint and life-cycle greenhouse gas emissions compared to fossil fuel. Therefore, the incorporation of AI-based injection optimization with renewable DF mode operation validates substantial possibility to achieve sustainable, low-emission, and energy-efficient combustion, strengthening the environmental feasibility of hydrogen–Spirogyra biodiesel employment in future green transportation systems.

## Conclusions

This current study mainly focused on evaluating the combustion, performance, and emission characteristics of a CI engine fueled with Blend-2 at different IPs of 180, 200, 220, and 240 bar. The following conclusions can be drawn:

- ID declines with higher load, attributed to higher cylinder temperature and compressed air. Blend-2 (IP = 220) shows the lowest ID, representing superior combustion characteristics. CD rises with load, and the IP of 220 bar displays a slight upsurge compared to the IP of 240 bar. Blend-2 (IP = 220) at full load is 32.56°CA.
- PCP is highest for IP = 240 bar, indicating efficient combustion and rapid combustion. Blend-2 (IP = 240) at full load: 83.8 bar. HRR is highest for Blend-2 (IP = 240) but 1.9% down for Blend-2 (IP = 220), demonstrating complete combustion and with an increase of 20 bar pressure doesn’t make much difference in the HRR.
- The BSFC for Blend-2 (IP = 220 bar) is 12.4% higher than Blend-2 (IP = 240 bar). The BTE for Blend-2 (IP = 220) is 18.4%, 35.1%, 12.0%, and 2.7% higher than diesel, SBD30, Blend-2 (IP = 180 bar), Blend-2 (IP = 200 bar), respectively at full load. EGT increases with load and is higher for H_2_ DF operation with increased IP.
- The lower CO emissions are significantly noted for H_2_ DF operation, particularly at IP = 240 bar. HC emissions reduce with load, and the IP of 240 bar displays lesser HC emissions. The increase in NO emissions increase with IP; Blend-2 (IP = 220) displays lesser NO emissions. Smoke emissions are lower for H_2_ DF operation, with IP = 240 bar showing the least smoke.
- The decision tree (DT) model reaches the maximum accurateness among all parameters. It yields test R² values of 0.9792 for PCP and 0.9710 for HC, accompanied by minimal MAPE values of 0 for BTE and HC. The Wi values exceed 0.99, finding it as the most reliable model for predicting performance and emissions.

The investigation suggests that Blend-2 at an IP of 220 bar is a promising blend, offering improved combustion efficiency, enhanced performance, and reduced emissions, making it a noteworthy candidate for further research and practical applications in diesel engines.

## Limitations of the work

Although the study successfully demonstrates the benefits of hydrogen-Spirogyra biodiesel dual-fuel operation with optimized injection pressure, some limitations remain. The experiments were conducted on a single-cylinder engine, which may not fully represent multi-cylinder commercial engines. The hydrogen flow rate was fixed at 15 LPM, preventing evaluation of the influence of variable hydrogen induction. The study focused only on four discrete injection pressures, leaving scope for finer optimization. Long-term durability, injector wear, carbon deposition, and safety aspects of hydrogen handling were not assessed. Machine learning predictions used a limited dataset, which may affect generalization accuracy under broader operating conditions. Fuel preparation and characterization of nano-additives were based on laboratory-scale procedures, which may differ when scaled to industrial production.

## Future scope and applications aligning with SDGs

The integration of hydrogen with Spirogyra biodiesel represents a promising pathway toward low-carbon, high-efficiency fuel systems that the world urgently needs. Future research can focus on scaling this dual-fuel technology to multi-cylinder, heavy-duty, and off-road engines, enabling broader adoption in transportation, agriculture, and decentralized power generation. Expanding machine learning–based optimization using larger datasets, real-time adaptive control, and digital twins will support intelligent combustion management and wider industrial integration. Additionally, advancing green hydrogen production and large-scale algae cultivation can create a fully renewable fuel cycle, reducing dependence on fossil fuels and lowering life-cycle emissions. Safety, storage, and fuel delivery systems for hydrogen can also be improved to enhance commercialization potential.

In terms of global applications, this dual-fuel system is highly relevant for countries seeking to decarbonize hard-to-electrify sectors, reduce petroleum imports, and strengthen energy security. Algae-based biodiesel production supports rural employment, CO₂ recycling, and waste-water utilization, enabling sustainable bio-resource management. When scaled, the technology can significantly benefit public transport fleets, marine engines, generators, and industrial power units requiring cleaner and more efficient combustion.

This work directly supports multiple UN Sustainable Development Goals (SDGs): SDG 7-Affordable and Clean Energy: Provides a renewable, efficient, and clean-burning alternative to fossil diesel. SDG 9-Industry, Innovation, and Infrastructure: Promotes smart engine technologies through AI-based optimization and advanced fuel systems. SDG 12-Responsible Consumption and Production: Encourages sustainable biomass utilization and renewable fuel cycles. SDG 13-Climate Action: Reduces life-cycle carbon emissions, NOx, smoke, and fossil-fuel dependence. SDG 14 & 15-Life Below Water and Life on Land: Microalgae cultivation reduces environmental burden and enhances CO₂ capture without competing for agricultural land.

Overall, hydrogen-enriched biodiesel, supported by AI-driven injection optimization, offers a scalable and globally relevant pathway towards cleaner mobility and sustainable energy systems for the future.

## Data Availability

The datasets used and/or analyzed during the current study are available from the corresponding author upon reasonable request.

## Abbreviations

H_2_: Hydrogen
DF: Dual-fuel
IP: Injection pressure
ID: Ignition delay
PCP: Peak cylinder pressure
HRR: Heat release rate
BSFC: Brake-specific fuel consumption
BTE: Brake thermal efficiency
EGT: Exhaust gas temperature
NOx: Nitrogen oxides
CO: Carbon monoxide
DT: Decision tree
R^2^: Coefficient of determination
MAPE: Mean absolute percentage error
Wi: Wilmott’s index
LR: Linear regression
RF: Random forest
bTDC: Before top dead center
DI CI: Direct injection compression ignition
CRDi: Common rail direct injection
IMEP: Indicated mean effective pressure
ML: Machine learning
CV: Calorific value
ARCNP: Advanced renewable catalytic nano particles
DTBP: Di-tert-butyl peroxide
ASTM: American Society for Testing and Materials
